# A zero-parameter first-principles gate framework for full-length TP53 missense variant interpretation

**DOI:** 10.1371/journal.pcbi.1014168

**Published:** 2026-06-11

**Authors:** Masamichi Iizumi

**Affiliations:** Miosync, Inc., Tokyo, Japan; Université de Montréal Faculté de médecine: Universite de Montreal Faculte de medecine, CANADA

## Abstract

Missense variant interpretation often achieves useful predictive performance but remains mechanistically opaque, particularly in proteins that combine structured domains with intrinsically disordered regions (IDRs). We developed Gate & Channel, a zero-parameter, first-principles framework for full-length TP53 missense variant analysis in which each prediction is generated by explicit IF-THEN gates derived from physicochemistry, geometry, structural constraints, and polymer physics rather than fitted weights. Variants are evaluated across independent channels representing distinct physical failure modes; a variant is predicted disruptive if any gate closes. A second hierarchical layer (“Geta”) encodes physically grounded post-closure exceptions, allowing sensitivity and specificity to be improved on disjoint variant populations.

The v18 framework consists of 12 channels and 2 Getas spanning structured domains and IDRs, capturing DNA-contact disruption, Zn coordination, burial-dependent packing, secondary-structure compatibility, post-translational modification chemistry, short linear motif disruption (including a multi-partner coupled-folding face), proline-directed kinase recognition, and IDR-specific proline and glycine backbone constraints. Across 1,369 TP53 missense variants, the framework achieved **84.5% sensitivity and 89.1% positive predictive value**, with 90.9% sensitivity preserved in the DNA-binding core and all 9/9 hotspot mutations captured. A post hoc audit of discordant IDR calls indicated that many apparent false positives had plausible molecular rationales, consistent with a distinction between molecular mechanism disruption and clinical penetrance.

Applied to KRAS, TDP-43, and BRCA1, the same channels capture the dominant pathogenic mechanisms in each protein as a proof of principle, while residual missed variants name specific gates yet to be written. The framework is distributed as the open-source Python package pathogenicity-gates (v0.5.1, MIT). These results show that a substantial fraction of full-length TP53 missense variation can be resolved through explicit, auditable physical gates that carry meaning beyond TP53, with each remaining failure naming the next rule to be written.

## Author summary

Most tools that predict whether a TP53 mutation is disease-causing rely on machine learning trained on large datasets. These models can be accurate but typically cannot explain *why* a particular mutation is flagged — which specific physical rule is broken. I developed a framework called Gate & Channel that takes a different approach: instead of learning patterns from data, it encodes explicit physical rules, such as “this mutation destroys a zinc-binding site.” If any single rule is violated, the mutation is flagged as disruptive. Applied across the full length of p53, including its intrinsically disordered regions, the framework correctly identified **nearly 85% of known disease-causing mutations with zero fitted parameters**, with a specific physical explanation for every call. The same rules captured the dominant pathogenic mechanisms in three other cancer-relevant proteins (KRAS, TDP-43, BRCA1); where they fail, the failure itself names the specific physical rule that needs to be added next. In this view, the framework is not a model of biology but a transcription of it: the physical laws have been established by generations of structural biologists, biochemists, and polymer physicists, and what remains is the patient work of writing them out as IF-THEN rules — one mechanism at a time.

## Introduction

Interpreting the consequences of missense variation remains a central problem in molecular biology and genomic medicine. In many genes, the key challenge is not only whether a variant is likely to be disruptive, but which physical constraint is being violated and how that violation propagates to molecular function. This problem is especially acute for TP53, whose full-length protein combines multiple physical regimes within a single sequence: an ordered DNA-binding core domain, a tetramerization domain, and extensive intrinsically disordered regions (IDRs) that mediate coupled folding, protein interaction motifs, post-translational modification (PTM) logic, localization signals, and regulatory charge patterns [1,2,3]. A single predictive formalism must therefore span coordination chemistry, steric packing, electrostatics, secondary-structure constraints, interface geometry, and polymer-like backbone behavior.

Many computational approaches to variant interpretation achieve useful performance by integrating large numbers of features through fitted weights, ensemble scoring, or learned latent representations [4–8]. Such models are valuable for ranking variants, but they often do not provide a direct mechanistic audit trail for any individual call. In practice, it may remain unclear whether a variant was classified because it disrupts a DNA contact, perturbs a metal-binding shell, creates a buried steric clash, abolishes a short linear motif, or simply resembles previously labeled variants in training space. This opacity becomes a particular limitation when the scientific goal is not only prediction, but physical explanation. A second limitation is that optimization against clinical labels can blur two distinct questions: whether a substitution disrupts molecular function, and whether that molecular disruption is sufficiently penetrant to appear as a pathogenic clinical label. For a mechanistic framework, these two levels should be kept conceptually separate.

To address this problem, we developed Gate & Channel, a zero-parameter, first-principles framework for full-length TP53 missense analysis. In this framework, each decision is written as an explicit IF-THEN gate derived from textbook physicochemistry, geometry, structural biology, or polymer physics. A channel groups gates that test a common mode of failure, such as DNA contact loss, Zn coordination disruption, core packing defects, PTM-site logic, or IDR motif disruption. A gate returns CLOSED when a specific physical constraint is violated; a variant is predicted disruptive if any gate in any channel closes. No weights are learned, no continuous risk score is fitted, and thresholds are not tuned against clinical labels. Instead, the framework is built around three design principles: thresholds should be derived from physical interpretation rather than label fitting, gates should be expressible as discrete IF-THEN rules rather than opaque composite scores, and missed variants should be interpreted as evidence of missing physical gates rather than overly strict thresholds.

We applied this framework to the full length of TP53, extending earlier structure-centered versions to include IDR-specific logic and a hierarchical post-closure exception layer. The resulting v18 framework combines structure-based gates for the ordered core and tetramerization regions with one-dimensional gates for the N-terminal transactivation/proline-rich segments, the linker, and the C-terminal regulatory tail. In addition to canonical structured-domain constraints, the full-length formulation introduces explicit gates for coupled folding at the multi-partner transactivation-domain interface (MDM2, CBP TAZ2/TAZ1/NCBD, and p300 TAZ2) [9], polyproline-II (PPII) incompatibility and spacer disruption in the proline-rich domain, proline-directed kinase recognition of [S/T]-P motifs [10], sequence-local PTM proximity rules that differ between structured and disordered regions, nuclear localization signal disruption, regulatory-tail charge-pattern perturbation, and IDR glycine constraints that formalize backbone freedom as function. A second architectural layer (“Geta”) encodes physically grounded post-closure exceptions, enabling sensitivity and specificity to improve on disjoint variant populations without lowering any threshold. Across 1,369 missense variants in modeled regions, the framework achieved an overall sensitivity of 84.5% and a positive predictive value of 89.1%, while preserving core-domain sensitivity at 90.9% despite the expansion to full-length coverage. More importantly, it produced explicit molecular rationales for individual variants, including variants in IDRs that are difficult to localize within a conventional structure-based model. The same rules were further evaluated on KRAS, TDP-43, and BRCA1, where they capture the dominant pathogenic mechanisms in each case and where the residual missed variants name specific gates yet to be written rather than performance failures.

This work was motivated by a simple premise: a computational predictor should not merely imitate labels, but encode the physical reasons why a substitution is expected to fail. In that sense, Gate & Channel is not designed as a black-box alternative to mechanistic reasoning; it is a computational formalism for mechanistic reasoning itself. By expressing missense interpretation as a collection of discrete, auditable, symmetry-aware physical gates, the framework provides both a full-length analysis of TP53 and a template for interpretable variant analysis in other multidomain proteins. The framework is distributed as the open-source Python package pathogenicity-gates (v0.5.1, MIT license) with a command-line interface and bundled annotations for p53, KRAS, TDP-43, and BRCA1; adding a new target protein requires only a YAML annotation file and a PDB or AlphaFold structure.

## Materials and methods

### Framework design principles

The Gate & Channel framework was constructed under four explicit design principles. First, thresholds were derived from physical interpretation rather than tuned against clinical labels. Second, each decision rule was required to be expressible as an IF-THEN gate representing a specific physical constraint. Third, when the framework failed to capture a pathogenic variant, the default interpretation was not that thresholds should be relaxed, but that an additional physical mechanism had not yet been encoded as a gate. Fourth, when expanding a channel to capture additional pathogenic variants produced incidental closures on legitimately tolerated variants, the framework added physically grounded post-closure exceptions (“Getas”) rather than weakening the underlying gate. In practice, this produced a deliberately discrete architecture: the model was not trained by regression, did not assign fitted channel weights, and did not optimize cutoffs on ClinVar [11].

**Fig 1:**
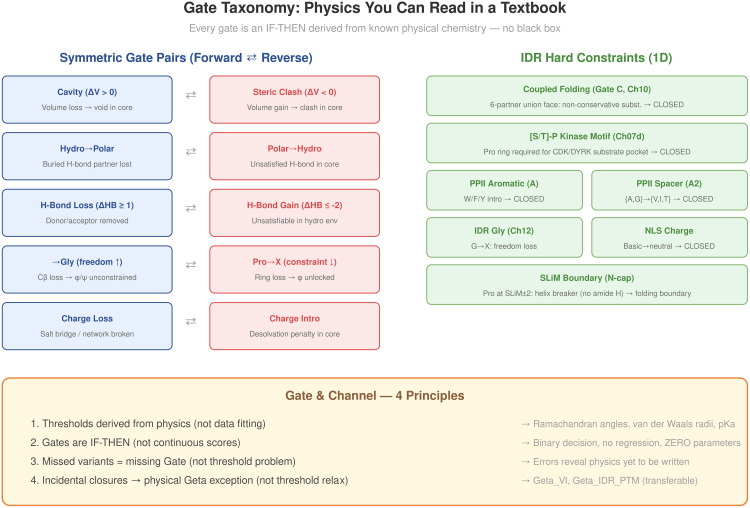
Gate taxonomy and symmetry logic of the Gate & Channel framework. The framework organizes gates into discrete physical categories, including hard backbone or chemical constraints, symmetric physicochemical perturbation pairs, and context-dependent functional gates. Mirror relations include cavity versus steric clash, hydrophobic-to-polar versus polar-to-hydrophobic shifts, hydrogen-bond loss versus gain, and complementary backbone endpoint constraints associated with glycine and proline. This symmetry-aware design reduces one-sided heuristics and constrains the model to explicit, auditable physical rules rather than fitted composite scores.

A second guiding principle was symmetry completeness wherever the underlying physics was bidirectional. For example, loss of side-chain volume in a buried environment and gain of side-chain volume in a buried environment were treated as mirror problems (cavity versus steric clash), as were charge loss versus charge introduction, hydro-to-polar versus polar-to-hydrophobic shifts, and hydrogen-bond loss versus hydrogen-bond gain. This avoided one-sided heuristics in which only one direction of a physically paired perturbation is represented. The gate taxonomy and symmetry logic of the framework are summarized in Fig 1.

### Input data and residue scope

The framework combined structural, sequence, and annotation inputs. Ordered-domain gates were derived from experimentally resolved structures. The p53 core DNA-binding domain was modeled from 1TSR [12], using chain B for the protein and DNA chains E/F for protein–DNA distance calculations. The tetramerization domain was modeled from 2J0Z [13]. The coupled-folding transactivation-domain interface was derived from a 6-partner union of complex structures: 1YCR (p53 TAD × MDM2) [9], 5HPD and 2K8F (p53 TAD × CBP TAZ2), 5HOU (p53 TAD × CBP TAZ1), 2L14 (p53 TAD2 × p300 TAZ2), and 2MZD (p53 TAD2 × NCBD), yielding a 59-residue union face spanning TAD1 and TAD2. Post-translational modification sites were taken from UniProt P04637 [14], yielding 31 annotated PTM positions across the full-length protein. Protein–protein interface context in the core domain was compiled from a union of 16 p53 complex structures, resulting in a 66-residue interface set.

Per-residue structural context was computed using the SSOC v3.32 framework and fixed amino-acid property tables for hydrophobicity, side-chain volume, charge, hydrogen-bond donor/acceptor capacity, aromaticity, sulfur content, helix propensity, and beta-sheet propensity. These quantities were treated as fixed physicochemical descriptors and were not re-estimated for TP53. For structured regions, the framework computed secondary structure, local contact density, burial, local polarity fraction, helix and beta neighborhood fractions, aromatic and sulfur-rich local environments, charged-neighbor density, glycine virtual Cβ placement, and related geometric quantities. Heavy-atom distances were used unless otherwise specified.

The full-length analysis covered three major physical regimes: the core DNA-binding domain (residues 94–289), the tetramerization region (residues 325–356), and intrinsically disordered segments in the N-terminus (residues 1–93), linker (residues 290–324), and C-terminal regulatory tail (residues 357–393). Ordered regions were analyzed with three-dimensional gates derived from experimental coordinates, whereas IDR segments were analyzed with one-dimensional gates derived from local sequence position, motif context, PTM proximity, and polymer-like backbone logic.

### Gate outputs, channels, and decision rule

Each variant was evaluated independently across a set of channels, and each channel contained one or more gates. A gate returned CLOSED when a specified physical constraint was violated and OPEN otherwise. Some structured-domain gates also returned a PARTIAL state for audit purposes when a substitution was physically unfavorable but did not cross a hard closure threshold. The binary decision rule, however, depended only on CLOSED gates.

For a variant *v*, the total number of closed gates was defined as


$$\begin{array}{c} n_{closed}(v)=\sum_{g}1[g(v)=CLOSED] \end{array}$$ (1)

and the final prediction was


$$\begin{array}{c} \text{Pathogenic if }n_{closed}(v)\geq 1;\;\text{Benign/VUS otherwise.} \end{array}$$ (2)

Thus, the framework did not average evidence across channels and did not require concordance among multiple gates. A single physically sufficient violation was treated as enough to classify a variant as disruptive. This “any gate closes” rule was central to the framework and reflects the intended interpretation of missense failure as the consequence of violating at least one indispensable constraint.

**Fig 2:**
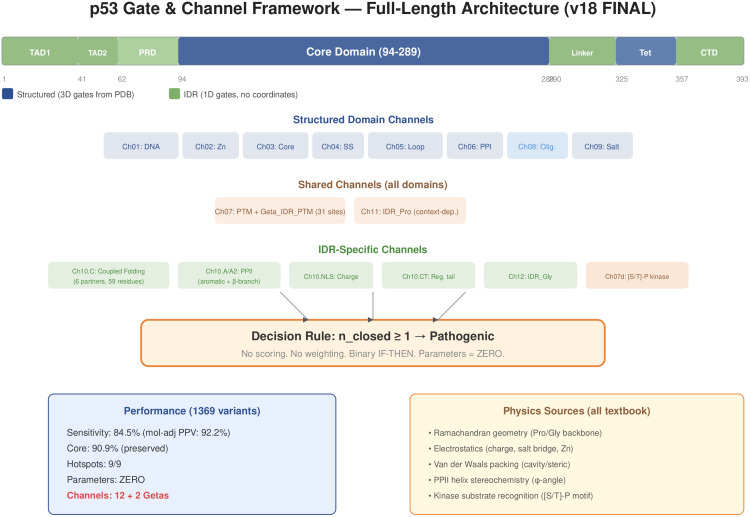
Overview of the full-length TP53 Gate & Channel framework. Schematic of TP53 domain organization across structured and intrinsically disordered regions and the mapping of Gate & Channel logic onto each region. Structure-based channels are applied to the DNA-binding core domain (1TSR) and tetramerization domain (2J0Z), whereas one-dimensional gates are applied to the N-terminal transactivation regions, the proline-rich domain, the linker, and the C-terminal regulatory tail. Each channel contains one or more binary gates that test a specific physical constraint, including DNA-contact geometry, Zn coordination, packing integrity, secondary-structure compatibility, PTM chemistry, short linear motif (SLiM) disruption, and IDR backbone constraints. A variant is predicted disruptive when one or more gates close (*n*_closed_ ≥ 1).

In v18 FINAL, the framework consists of 12 channels and 2 Getas: structured-domain channels Ch01_DNA (DNA contact), Ch02_Zn (Zn coordination), Ch03_Core (core integrity), Ch04_SS (secondary-structure compatibility), Ch05_Loop (loop and Pro/Gly Ramachandran logic), Ch06_PPI (protein–protein interface), Ch07_PTM (PTM-site logic, including structured- and IDR-specific sub-gates), Ch08_Oligomer (oligomeric interface; tetramer in p53), Ch09_SaltBridge (surface salt-bridge networks); IDR-specific channels Ch10_SLiM (SLiM motif logic, including the multi-partner coupled-folding face), Ch11_IDR_Pro (context-dependent IDR proline logic), Ch12_IDR_Gly (IDR glycine constraint); and two post-closure Getas, Geta_VI (V ↔ I isomeric exception in Ch03_Core) and Geta_IDR_PTM (charge-preserving PTM-proximity exception in Ch07_PTM IDR sub-gates). An overview of the full-length TP53 Gate & Channel architecture is shown in Fig 2.

### Hierarchical Gate–Geta architecture

A single-layer IF-THEN gate produces false positives when a physical condition for closure is satisfied but the variant is in fact tolerated due to a secondary physical context not captured by the primary condition. To prevent this without weakening the underlying gate, the framework introduces a second layer in the form of post-closure exceptions called *Getas*. The name is taken from the Japanese typesetting placeholder *geta* mark (the symbol used when a character is missing from a font); here it lifts a closed variant out of closure when secondary physics indicates tolerance.

Formally, a Geta is a function *G* that maps a CLOSED gate output back to OPEN if a secondary physical condition is satisfied:


$$\begin{array}{c} state(g,v)=\left\{ \begin{array}{cc} OPEN & \text{if }g(v)=CLOSED\text{ and }G(v)=TRUE,\\ g(v) & \text{otherwise.} \end{array}\right. \end{array}$$ (3)

A Geta is admitted into the framework only if all three of the following conditions hold. (i) Its condition is expressible in protein-independent physicochemical quantities (volume, charge, hydrophobicity, β-branching, burial, IDR/PTM annotation), and does not refer to ClinVar labels, variant frequencies, or statistical priors. (ii) When evaluated across all ClinVar Pathogenic variants, the Geta rescues zero of them. (iii) Its condition generalizes to any protein where the primary gate applies (no residue numbers, no protein-specific partner identities).

Two Getas are used in v18 FINAL. Geta_VI applies to Ch03_Core: when the primary gate closes and the substitution is V ↔ I, the closure is reversed. The physical justification is that V and I differ by a single methylene, are both β-branched and similarly hydrophobic, and are therefore mutually compatible with buried positions that admit either rotamer. Geta_IDR_PTM applies to the IDR sub-gates of Ch07_PTM: when the primary gate closes within ±1 residue of a PTM site and the substitution is one of K ↔ R, R ↔ H, or S ↔ T, the closure is reversed. The physical justification is that disordered backbones absorb local steric and electrostatic perturbations and that these specific swaps preserve charge and hydrogen-bond chemistry; they therefore do not disrupt enzyme recognition. Both Getas were verified to rescue zero ClinVar Pathogenic variants. We emphasize that the Getas were proposed by inspecting individual false positives, not by aggregate optimization; this corresponds to a methodological commitment to address each FN and FP as an individual case rather than by parameter adjustment.

**Fig 3:**
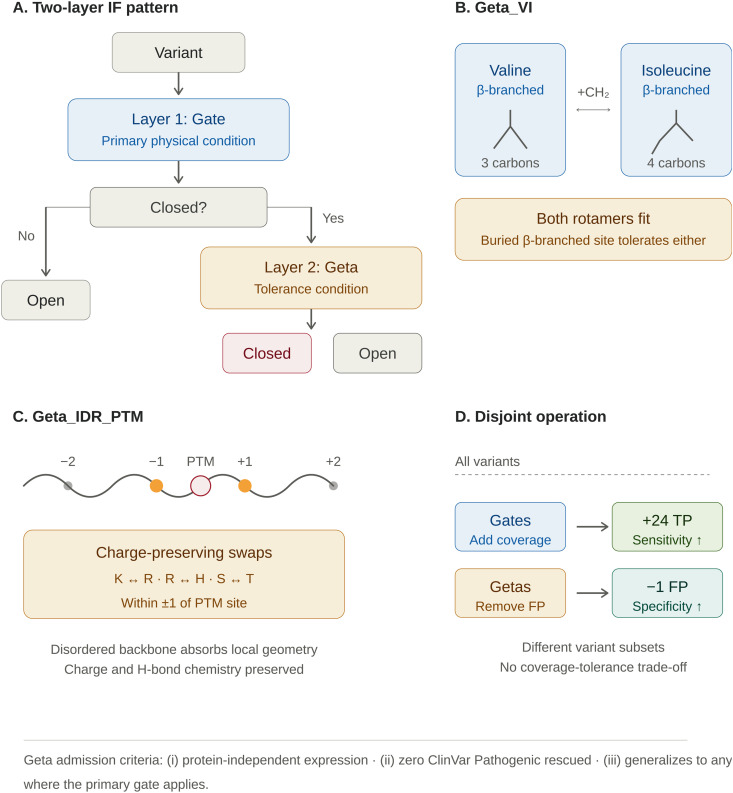
Hierarchical Gate–Geta architecture. A: Two-layer IF pattern. Layer 1 (Gate) closes when a primary physical condition is satisfied. Layer 2 (Geta) reverses the closure if a secondary physical condition indicates legitimate tolerance. A Geta is admitted only if (i) it is expressible in protein-independent physicochemical quantities, (ii) it rescues zero ClinVar Pathogenic variants, and (iii) it generalizes to any protein where the primary gate applies. Condition (ii) was used as a post hoc safeguard against reversing known disruptive variants, not as a fitting objective or threshold-optimization step. B: Geta_VI rescues V ↔ I substitutions in Ch03_Core: both residues are β-branched aliphatic and differ by one methylene, so buried positions admitting one rotamer admit the other. C: Geta_IDR_PTM rescues charge-preserving substitutions (K ↔ R, R ↔ H, S ↔ T) within ±1 residue of a PTM site in IDR regions: disordered backbones absorb local geometric perturbations, and these specific swaps preserve charge and hydrogen-bond chemistry. D: Disjoint operation. Gates and Getas act on different variant populations, so coverage (sensitivity) and tolerance (specificity) can be improved together rather than traded against each other.

Because Gates and Getas act on disjoint variant populations — Gates expand coverage and add true positives, Getas remove specific false positives — the two layers are approximately independent and can be developed separately. This is the architectural reason that v18 FINAL improves both sensitivity and specificity (relative to a hypothetical Gate-only expansion) on different variant subsets. The architecture is summarized in Fig 3.

### Structured-domain channels

#### Ch01_DNA: DNA-contact disruption.

DNA-contact gates were evaluated in the core domain using the minimum distance between protein side-chain heavy atoms and DNA heavy atoms in 1TSR. Two distance regimes were used. A direct-contact regime captured residues with side-chain–DNA distances below 3.5 Å, where disruption of basicity, hydrogen-bond capacity, or charge was sufficient to close the gate. A proximal-contact regime extended to 6.0 Å and captured substitutions expected to weaken the local electrostatic or hydrogen-bonding environment around the DNA interface. This channel formalized the idea that DNA recognition is governed not merely by residue identity, but by short-range side-chain geometry and chemistry at the bound interface.

#### Ch02_Zn: Zn-coordination logic.

Zn coordination was modeled as a layered cascade. The direct ligand positions C176, H179, C238, and C242 were treated as hard-closure sites. In addition, residues within the electrostatic environment of Zn were evaluated by protein Cα-to-Zn distance and charge change. Substitutions within 8 Å of Zn that introduced a sufficiently large charge perturbation closed the gate. This channel separated direct coordination chemistry from disruption of the surrounding electrostatic shell.

#### Ch03_Core: core integrity and symmetry-complete packing logic.

The core-integrity channel encoded burial-dependent constraints on packing, polarity, hydrogen bonding, aromaticity, sulfur-rich environments, and electrostatics. A set of Tier S hard constraints captured physically severe events such as buried X → Gly substitutions (interpreted as Ramachandran freedom explosion), loss of sulfur-mediated S⋯π or sulfur-rich local networks, loss of β-branching in buried β-strands, and buried polar-to-hydrophobic substitutions that remove hydrogen-bond compatibility.

Beyond these hard constraints, the channel used symmetry-complete packing logic. Volume loss and volume gain were represented as mirror perturbations corresponding to cavity formation and steric clash, respectively, modulated by burial and local void propensity. Additional mirror gates captured charge introduction versus charge loss, sign reversal of buried charge, hydrophobic-to-polar versus polar-to-hydrophobic shifts, hydrogen-bond loss versus gain, and aromatic gain in buried sites. The channel also included a surface hydrophobic exposure rule for solvent-exposed hydrophobic residues and an electrostatic-keystone rule for residues embedded in dense charged networks. By construction, this channel avoided treating only one direction of a paired physical perturbation as meaningful.

#### Ch04_SS: secondary-structure disruption.

Secondary-structure gates tested whether a substitution was incompatible with the local helix or β-strand environment. In helices, closure could be triggered by sufficiently large decreases in helix propensity, backbone-strain penalties, or proline disruption at helix cores or helix-defined proline positions. In β-strands, closure could be triggered by sufficient loss of β-propensity, proline loss in β-structure, or strong strand-incompatible substitutions in buried β-neighborhoods. This channel formalized local backbone and secondary-structure compatibility rather than global fold change.

#### Ch05_Loop: structured loop and Pro/Gly Ramachandran constraints.

The structured-domain loop channel implemented Ramachandran-centered logic for glycine and proline. Glycine-to-non-glycine substitutions were captured in the major loop regions L2/L3 and in other contexts where glycine-specific backbone freedom was structurally required. Non-proline-to-proline substitutions closed the gate in helices, β-strands, and loop contexts known to be sensitive to proline insertion. Conversely, Pro → X in structured regions was treated as a hard closure because release of proline-imposed backbone constraint was considered physically severe in ordered local structure. Additional loop-anchor positions were evaluated for large charge or volume perturbations.

#### Ch06_PPI: protein–protein interface disruption.

Protein–protein interface gates were derived from the union of 16 p53 complex structures. This channel was restricted to residues represented in the interface union and emphasized experimentally recurring contact geometry. A gate closed when a residue was observed in multiple complexes and underwent a sufficiently large physicochemical change, when direct intermolecular contact distances were short enough that charge or hydrophobicity changes were expected to disrupt the interface, or when an interface-core residue with dense neighboring interface context underwent substantial charge or volume change. The goal was to capture interface disruption from resolved interaction geometry rather than from generic surface heuristics.

#### Ch07_PTM: PTM-site logic in structured regions.

PTM logic was split into multiple sub-gates. A direct-hit gate closed when a residue at an annotated PTM site lost the expected side-chain chemistry required for that modification. In structured regions, a second gate captured substitutions within a short local radius (±2 residues) of a PTM site when they introduced a sufficiently large charge or volume perturbation. This gate used OR logic rather than requiring simultaneous charge and size effects, because electrostatic recognition and steric accessibility were treated as independent mechanisms.

#### Ch08_Oligomer: oligomer-interface disruption (tetramer in p53).

Tetramerization was modeled from 2J0Z using interface distance, exposure asymmetry, and multi-chain context. Variants at tetramer-interface residues were evaluated by the magnitude of physicochemical demand imposed by the substitution together with local interface exposure. A separate rigid hub rule closed the gate when a residue contacted two or more partner chains simultaneously, representing positions whose geometry could not be compensated by a simple single-interface rearrangement.

#### Ch09_SaltBridge: surface salt-bridge disruption.

Surface salt-bridge logic identified charged core-domain residues with oppositely charged partners within 10 Å under surface-exposed conditions. A sufficiently large charge change at such positions closed the gate. This channel treated solvent-exposed electrostatic “zippers” as hard physical constraints rather than as generic surface features.

### IDR-specific channels

#### One-dimensional representation of disordered regions.

IDR segments were not treated as lacking structure in a trivial sense; instead, they were modeled as regions whose function is encoded by local sequence-dependent constraints rather than a single stable tertiary conformation [2,15]. Accordingly, IDR gates were one-dimensional and operated on motif identity, motif boundaries, PTM proximity, local physicochemical change, and backbone freedom. This allowed the framework to express coupled folding, sequence motif recognition, and polymer-like chain behavior without fitting statistical sequence models.

#### Ch11_IDR_Pro: context-dependent IDR Pro→X logic.

In IDRs, Pro → X was not treated as a universal hard constraint. Instead, proline loss was evaluated according to context. Closure was assigned when proline was required for the polyproline-rich domain, when it occurred within a defined SLiM interior, when it was positioned at or near a motif boundary where proline acts as a folding delimiter or helix breaker [16], or when it lay near a PTM site where backbone geometry was likely to affect enzyme access. By contrast, isolated prolines lacking defined local functional context were left open. The common polymorphic P72 site was explicitly excluded from closure in this channel [17].

#### Ch12_IDR_Gly: IDR glycine constraint.

A separate IDR glycine gate encoded the principle that, in disordered regions, backbone freedom can itself be functional. Glycine-to-non-glycine substitutions were therefore treated as hard closures across IDR segments, based on the interpretation that introducing a side chain constrains the conformational space required for coupled folding, flexible linker behavior, or PTM accessibility.

#### Ch07_PTM IDR sub-gates: MECE PTM logic and proline-directed kinase motifs.

PTM logic in IDRs was split from structured-region logic in a mutually exclusive and collectively exhaustive (MECE) manner. Direct PTM-site hits were handled as above, but proximity rules differed between ordered and disordered regions. In structured regions, perturbation within a short ±2 residue window around a PTM site was considered sufficient to transmit steric or electrostatic disruption. In IDRs, the corresponding proximity rule was restricted to the immediate ±1 residue neighborhood, reflecting the assumption that a flexible backbone absorbs longer-range local perturbations more readily than a rigid structured segment.

A further PTM sub-gate captured proline-directed kinase motifs of the form [S/T]-P. When a proline immediately following an annotated phosphosite was replaced, the gate was closed if the site was recognized by a proline-directed kinase family [10]. This gate treated the cyclic proline ring itself as the recognition element, rather than treating the event as a generic physicochemical substitution.

#### Ch10_SLiM: SLiM motif disruption (including the multi-partner Gate C coupled-folding face).

The SLiM channel grouped several distinct IDR mechanisms. First, a coupled-folding interface gate (*Gate C*) was derived from a 6-partner union of complex structures: 1YCR (MDM2) [9], 5HPD and 2K8F (CBP TAZ2), 5HOU (CBP TAZ1), 2L14 (p300 TAZ2 with TAD2), and 2MZD (NCBD with TAD2). The union face spans 59 residues across TAD1 and TAD2; substitutions at any union-face position were treated as candidate disruptions. To prevent over-closure, Gate C closure required additionally that the substitution be non-conservative (|ΔQ|≥0.3 or |ΔV|≥30 Å^3^ or |Δh|≥1.5 on the hydrophobicity scale), reflecting that coupled folding can accommodate side-chain chemistry-preserving rotamer adjustments via induced-fit plasticity. Second, the proline-rich domain was analyzed for PPII incompatibility [18], including introduction of bulky aromatic residues incompatible with PPII backbone geometry and β-branched spacer substitutions expected to restrict the backbone angles required for polyproline-II structure. Third, nuclear localization signals were represented by charge-dependent gates at critical basic positions [19], such that loss of basicity at those sites closed the gate. Fourth, aromatic anchor residues in short binding motifs were modeled explicitly. Finally, the C-terminal regulatory region was assigned a charge-pattern gate so that sufficiently large charge perturbations in that tail were treated as disruptive to regulatory electrostatics.

### AlphaFold compatibility for proteins lacking experimental coverage

The framework’s coordinate-dependent channels (Ch03_Core, Ch04_SS, Ch05_Loop, Ch06_PPI, Ch09_SaltBridge, and the Tier-S sub-gates of Ch03) compute burial, secondary structure, contact density, and charge-network proximity from atomic coordinates. For protein regions where AlphaFold [20] predictions exhibit pLDDT > 70, the predicted backbone is expected to be sufficiently accurate for the geometric quantities used in the present framework; the framework therefore accepts AlphaFold-derived structures in this regime without modification. The IDR-specific channels (Ch10_SLiM, Ch11_IDR_Pro, Ch12_IDR_Gly, and the IDR sub-gates of Ch07_PTM) are one-dimensional and operate on sequence and annotation alone; AlphaFold’s well-known low confidence in disordered regions (pLDDT typically < 50) is therefore not a constraint on this framework, and the 1D design of these channels was chosen specifically to bypass it. Coupled-folding interfaces (Gate C within Ch10_SLiM) can be extended to partners without experimental complex structures using AlphaFold-Multimer [21] predictions; the interface-residue-extraction pipeline that produces the union face accepts experimental or predicted complexes interchangeably, with appropriate confidence filters.

### Post hoc evaluation against clinical labels

Clinical labels were used only for post hoc evaluation and were not used to fit thresholds or coefficients. Missense variants from ClinVar [11] were filtered to in-scope TP53 positions covered by the full-length framework. Sensitivity, specificity, positive predictive value, and negative predictive value were computed using ClinVar pathogenic and benign labels, whereas variants labeled as uncertain significance were tracked separately as candidate disruptive variants when one or more gates closed. Canonical hotspot capture was also monitored as a diagnostic readout of coverage [22].

Importantly, disagreement with clinical labels was not interpreted uniformly as model failure. At the case level, discordant variants were audited mechanistically to distinguish likely missing gates from cases in which the framework identified a plausible molecular disruption not fully reflected by a penetrance-based clinical classification. This interpretation was particularly relevant in IDRs, where biochemical mechanism and clinical epidemiology may diverge. Per-variant classification results and gate-level outputs are provided as S1 Table.

## Results

### Full-length extension preserves structured-domain performance while expanding mechanistic coverage across TP53

We first asked whether the Gate & Channel framework could be extended from the ordered core of p53 to the full-length protein without introducing fitted parameters or sacrificing performance in the canonical structured domains. Evaluated against ClinVar labels, the v18 FINAL framework yielded 547 true positives, 67 false positives, 100 false negatives, and 67 true negatives across 1,369 in-scope variants, corresponding to a sensitivity of 84.5%, a specificity of 50.0%, and a positive predictive value (PPV) of 89.1% (Table 1). All nine canonical hotspot mutations were captured, and no parameters were fitted to ClinVar labels. In addition, 407 of 578 ClinVar variants of uncertain significance (70.4%) were flagged as candidate disruptive by one or more closed gates, consistent with the intended use of the framework as a mechanistic screening system rather than a penetrance-trained clinical classifier.

**Table 1:** Full-length p53 Gate & Channel v18 FINAL classification performance. Variants were classified using ClinVar pathogenic and benign labels. Ten variants with other or conflicting ClinVar significance were excluded from the binary (TP/FP/FN/TN) analysis but included in the total count.

Metric	Value
Variants evaluated	1,369
Pathogenic (ClinVar)	647
Benign (ClinVar)	134
VUS (ClinVar)	578
Other/Conflicting	10
True positives (TP)	547
False positives (FP)	67
False negatives (FN)	100
True negatives (TN)	67
Sensitivity	84.5%
Specificity	50.0%
PPV	89.1%
NPV	40.1%
Hotspots captured	9/9
VUS reclassified	407/578 (70.4%)
Fitted parameters	0

Importantly, full-length expansion did not degrade structured-domain performance. In the DNA-binding core, sensitivity remained 90.9% with a PPV of 95.4%, indicating that the addition of IDR-specific logic and the Gate–Geta layer did not erode the performance of the earlier structure-based framework (Table 2). Performance in the tetramerization region was lower but remained interpretable, with a sensitivity of 67.5% and a PPV of 71.1%. Thus, the extension to full-length TP53 preserved the strongest structured-domain signal while increasing the scope of mechanistic coverage.

**Table 2:** Per-region classification performance. Gate type indicates whether three-dimensional (3D) structural gates or one-dimensional (1D) sequence-based gates were applied. Sensitivity and positive predictive value are computed over ClinVar pathogenic and benign labels only.

Region	Gate	N	Path	Ben	VUS	TP	FP	FN	TN	Sens%	PPV%
Core (94–289)	3D	821	453	40	320	412	20	41	20	90.9	95.4
Tet (325–356)	3D	106	40	18	46	27	11	13	7	67.5	71.1
TAD1 (1–40)	1D	89	38	11	40	35	7	3	4	92.1	83.3
TAD2 (41–61)	1D	57	21	9	27	16	7	5	2	76.2	69.6
PRD (62–93)	1D	98	30	17	51	20	6	10	11	66.7	76.9
Linker (290–324)	1D	99	31	22	46	13	6	18	16	41.9	68.4
CTD (357–393)	1D	99	34	17	48	24	10	10	7	70.6	70.6
IDR total	1D	442	154	76	212	108	36	46	40	70.1	75.0

Performance across intrinsically disordered regions was heterogeneous, as expected for a regime in which local motifs, PTM logic, and backbone constraints replace stable tertiary structure. The two transactivation domains were captured well (TAD1 92.1%, TAD2 76.2%), driven by the multi-partner Gate C face derived from the union of 1YCR, 5HPD, 5HOU, 2L14, 2K8F, and 2MZD (MDM2 + CBP TAZ2/TAZ1/NCBD + p300 TAZ2). The proline-rich domain (PRD), the C-terminal domain (CTD), and the tetramer linker captured a sensitivity range of 41.9–70.6%, with the linker as the lowest. When aggregated across all disordered regions, the IDR-specific channels achieved a sensitivity of 70.1% and a PPV of 75.0% (Table 2). The regional pattern is informative: regions where partner-complex structures and motif annotations are present are well-captured, while the linker — which lacks defined SLiM and partner annotation — names a specific gap to be filled by additional binding or dynamics data.

The framework also preserved the expected mechanistic decomposition of classical p53 hotspot variants. Zn-centered structural lesions such as R175H, C176F, and H179R were captured by Ch02_Zn, with some variants additionally closing Ch03_Core or Ch05_Loop gates. DNA-contact hotspots such as R248W, R273H, and R280K were captured by Ch01_DNA, whereas Y220C and G245S were captured primarily by Ch03_Core logic (Table 3). Thus, complete hotspot capture was achieved not through a single global damage score, but through distinct channels that map directly onto known physical failure modes.

**Table 3:** Hotspot mutation capture (9/9). Each hotspot variant is listed with the channels that returned CLOSED. Multiple channels closing for a single variant reflect independent physical failure modes detected simultaneously.

Variant	Closed channels
R175H	Ch02_Zn, Ch03_Core, Ch05_Loop
C176F	Ch02_Zn, Ch03_Core, Ch05_Loop
H179R	Ch02_Zn
Y220C	Ch03_Core
G245S	Ch03_Core, Ch05_Loop
R248W	Ch01_DNA, Ch05_Loop, Ch06_PPI
R249S	Ch03_Core, Ch05_Loop
R273H	Ch01_DNA, Ch03_Core
R280K	Ch01_DNA, Ch03_Core

Together, these results show that v18 FINAL extends Gate & Channel from a structured-domain TP53 framework to a full-length model without sacrificing core-domain sensitivity, while exposing where additional IDR physics remains to be encoded.

### New IDR gates recover interpretable pathogenic signal from disordered regions

We next asked whether intrinsically disordered regions of TP53 could be analyzed through explicit one-dimensional physical gates rather than through fitted sequence statistics. Across all IDR segments, the dominant burden of signal was carried by three channel classes: SLiM-based motif logic (Ch10_SLiM, including the 6-partner Gate C), PTM-site and PTM-proximity logic (Ch07_PTM), and context-dependent proline logic in disordered regions (Ch11_IDR_Pro) (Table 4). These channels closed in 190, 224, and 92 variants, respectively, with PPVs of 74.3%, 76.5%, and 79.5%. A fourth channel, the IDR glycine constraint (Ch12_IDR_Gly), fired less frequently (24 variants) but captured a conceptually distinct mechanism: loss of functionally required backbone freedom.

**Table 4:** IDR and shared-channel firing statistics. Precision is computed over ClinVar pathogenic and benign labels only (VUS excluded from denominator). Ch07_PTM fires across all domains but is included here because its IDR sub-gates (7c, 7d) are central to the IDR extension.

Channel	CLOSED	Path	Ben	VUS	PPV
Ch10_SLiM (incl. Gate C)	190	75	26	89	74.3%
Ch07_PTM (all domains)	224	91	28	102	76.5%
Ch11_IDR_Pro	92	35	9	48	79.5%
Ch12_IDR_Gly	24	10	5	9	66.7%

These IDR gates encoded separable physical mechanisms rather than generic local sequence effects. The coupled-folding sub-gate represented the MDM2-binding face defined from the 1YCR complex and treated substitutions there as disruptions of bound-state interface geometry. The PPII incompatibility gate formalized steric incompatibility between aromatic residues and polyproline-II backbone geometry, whereas the PPII spacer β-branch gate captured a different mechanism, namely φ-angle restriction imposed by β-branched substitutions at small spacer positions in the PRD (Table 5). In parallel, the NLS sub-gate captured basic-residue loss at import-signal positions, the aromatic-anchor gate represented motif-specific hydrophobic packing, and the CT-regulatory gate formalized charge-pattern disruption in the C-terminal tail.

**Table 5:** Ch10 SLiM sub-gate physics. Each sub-gate captures a distinct physical mechanism operating in IDR motifs.

Sub-gate	Physical mechanism	Example variants
Gate C: Coupled folding	6-partner union face (1YCR/5HPD/5HOU/2L14/2K8F/2MZD)	F19X, W23X, L26X, F54X
Gate A: PPII incompatible	Aromatic steric clash with PPII backbone	X → W/F/Y in PRD
Gate A2: PPII spacer β-branch	Cβ di-substitution restricts φ-angle	A69V, A83V, A86V
Gate NLS: Charge loss	Basic residue loss abolishes import signal	R379C, K373N
Gate ARO: Aromatic anchor	W/F/Y identity required for binding pocket	W53L, F54L
Gate CT_reg: Charge pattern	Charge distribution controls regulation	D391N, T387R

One of the strongest new signals emerged from proline-directed kinase motifs. Across the three validated [S/T]-P sites represented by S33-P34, S46-P47, and S315-P316, all eight curated pathogenic Pro → X variants were captured by the framework (Table 6). This result supports a sharp mechanistic interpretation: in these motifs, the proline ring itself functions as part of the kinase recognition element, and its loss is therefore not a mild physicochemical substitution but destruction of the substrate motif.

**Table 6:** Proline-directed kinase [S/T]-P motif validation. All eight curated pathogenic Pro → X variants at validated motifs were captured. The cyclic imino-acid ring of proline functions as a kinase substrate-recognition element.

Phosphosite	Pro pos	Kinase	Pro → X variants	Result
S33	P34	CDK5/CDK7	P34A/T/R/S	All Path (4/4)
S46	P47	CDK5/DYRK2/HIPK2	P47T/R	Path (2/2)
S315	P316	CDK1/CDK2	P316L/S	Path (2/2)

A second motif-level discovery was the SLiM-boundary N-cap gate. At position P12, immediately N-terminal to the BOX_I region, substitutions were captured jointly by the IDR proline channel and the SLiM channel (Table 7). The physical rationale was that proline acts as a folding boundary because it lacks an amide hydrogen and therefore cannot participate in canonical i→i+4 helix hydrogen bonding. Under this model, loss of P12 removes a local helix-breaking delimiter and allows helix propagation beyond the intended coupled-folding boundary. The fact that both ClinVar-pathogenic and ClinVar-VUS substitutions at P12 were captured suggests that this gate identifies a real local physical constraint even where clinical interpretation remains incomplete.

**Table 7:** SLiM boundary N-cap gate at P12. Proline lacks an amide hydrogen and therefore cannot form i→i+4 backbone hydrogen bonds, acting as a helix-propagation boundary at the BOX_I N-terminus.

Variant	ClinVar	Prediction	Gates closed
P12L	Pathogenic	Pathogenic	Ch11_IDR_Pro, Ch10_SLiM
P12H	VUS	Pathogenic	Ch11_IDR_Pro, Ch10_SLiM
P12R	Pathogenic	Pathogenic	Ch11_IDR_Pro, Ch10_SLiM
P12S	VUS	Pathogenic	Ch11_IDR_Pro, Ch10_SLiM

Finally, Ch12_IDR_Gly formalized a principle absent from most structure-centered variant predictors: in intrinsically disordered regions, the absence of a side chain can itself be functional. The IDR glycine constraint therefore treated G → X substitutions as losses of backbone freedom rather than as conventional amino-acid replacements. Although this channel fired less often than the proline-, PTM-, and SLiM-based channels (24 variants; Table 4), it encoded an important conceptual point: disorder is not the absence of mechanism, but a regime in which conformational freedom is itself part of the mechanism.

Taken together, these results show that substantial pathogenic signal can be recovered from TP53 IDRs using discrete, auditable gates derived from PTM accessibility, local motif geometry, coupled folding, charge-pattern logic, and backbone freedom, without resorting to fitted black-box models.

IDR false negatives were not distributed randomly. Instead, they clustered into a small number of unresolved physical categories, including conservative substitutions likely requiring partner-complex geometry, non-proline PRD variants likely requiring SH3/interface identity, linker variants lacking defined motif or PTM context, CTD boundary variants around residues 360–362, and isolated IDR Pro → X substitutions suggestive of polymer-level constraints (S4 Table). This clustering supports the design principle that missed variants identify gates that have not yet been written, rather than overly strict thresholds.

### Post hoc audit of discordant IDR calls distinguishes likely model error from molecular–clinical decoupling

The lower specificity of the full-length framework in its standard ClinVar evaluation was concentrated in the disordered regions. We therefore asked whether all apparent IDR false positives reflected genuine gate errors — variants where the framework had identified the wrong physics — or whether some instead arose because Gate & Channel resolves molecular disruption whereas ClinVar labels reflect clinical penetrance.

To address this, we performed a post hoc mechanistic audit of the 36 IDR variants classified as Benign in ClinVar but predicted disruptive by one or more closed gates in v18 FINAL (Table 8). Of these, 19 had explicit, variant-level physical rationales consistent with true molecular disruption. These included substitutions near PTM sites that altered local charge or steric accessibility (P8L, P8R, D21A, R290C, G293W, D393Y), direct perturbation of chemically specific modification sites (K292R, S315T), destruction of import-signal basicity in the NLS (R379S, R379L, R379H), charge-pattern perturbation in the C-terminal regulatory region (E388A, E388Q, Q375K, G374R), and disruption of the multi-partner coupled-folding face of TAD2 captured by the v18 6-partner Gate C (D49H, D49N, I50T) (Table 9). The best-established precedent was P47S, which is classified as Benign in ClinVar but has been biochemically associated with reduced S46 phosphorylation and impaired apoptotic function [17]; in the framework, P47S closes both Ch11_IDR_Pro and Ch07_PTM, placing it naturally in the molecular-disruption category despite low clinical penetrance. The three new TAD2 entries identified in v18 (D49H, D49N, I50T) reflect the same logic on the partner-face side: D49 is a charged residue at the TAD2 amphipathic helix face that contacts p300 TAZ2 (2L14) and NCBD (2MZD); D → H reduces charge from −1 to approximately +0.1 at physiological pH (His pK_*a*_ ≈6), D → N removes the charge entirely, and I → T at the adjacent hydrophobic anchor I50 introduces a polar hydroxyl in place of a β-branched aliphatic side chain, disrupting both packing and desolvation in the coupled-folding interface. The complete TMP/Genuine-FP partition is given in Table 9 (TMPs) and Table 10 (Genuine FPs), with the audit summary in Table 8.

**Table 10:** Genuine false positives in IDR (v18 FINAL). These 17 variants do not yet have a sufficiently convincing physical rationale and are retained as next-gate targets. New v18 entries (bold) all sit at or near the multi-partner Gate C face; their resolution corresponds to specific direction-aware or partner-extension refinements that are scheduled for subsequent releases.

Variant	Region	Channels closed
**P4L**	TAD1	Ch10_SLiM (direction-aware Geta pending)
**P4Q**	TAD1	Ch10_SLiM (direction-aware Geta pending)
N29D	TAD1	Ch10_SLiM
P36Q	TAD1	Ch10_SLiM, Ch11_IDR_Pro
**D48G**	TAD2	Ch10_SLiM (Gate C union missing position 48)
**P58A**	TAD2	Ch10_SLiM (conservative substitution near threshold)
G59D	TAD2	Ch10_SLiM, Ch12_IDR_Gly
A74V	PRD	Ch10_SLiM
P75S	PRD	Ch10_SLiM, Ch11_IDR_Pro
A79V	PRD	Ch10_SLiM
P82L	PRD	Ch10_SLiM, Ch11_IDR_Pro
A83P	PRD	Ch11_IDR_Pro
A84T	PRD	Ch10_SLiM
G302A	Linker	Ch12_IDR_Gly
P316T	Linker	Ch07_PTM, Ch11_IDR_Pro
G360A	CTD	Ch12_IDR_Gly
T387P	CTD	Ch11_IDR_Pro

**Table 8:** IDR false-positive reclassification summary (v18 FINAL). The 36 IDR variants classified as Benign in ClinVar but predicted disruptive in v18 FINAL were audited for explicit physical rationales. R290H, listed as a True Molecular Positive in v17, is correctly rescued in v18 by Geta_IDR_PTM and therefore no longer appears in the FP list (see text).

Category	Count
Total IDR FP (ClinVar Benign, Gate CLOSED)	36
True Molecular Positives (physical rationale)	19
Genuine False Positives (no rationale)	17

**Table 9:** True Molecular Positive variants in IDR (v18 FINAL). Each variant has an explicit physical mechanism of molecular disruption identified by the framework despite ClinVar Benign classification. R290H (listed as TMP in v17) is correctly rescued in v18 by Geta_IDR_PTM and therefore no longer appears here. Three new TAD2 entries (D49H, D49N, I50T) were captured by the v18 6-partner Gate C expansion.

Variant	Channels closed	Physical mechanism
P8L	Ch07_PTM, Ch10_SLiM, Ch11_IDR_Pro	±2 to PTM [S9]: + 54 Å^3^ steric block
P8R	Ch07_PTM, Ch10_SLiM, Ch11_IDR_Pro	±2 to PTM [S9]: charge intro disrupts kinase recognition
D21A	Ch07_PTM, Ch10_SLiM	±2 to PTM [S20]: charge loss disrupts kinase recognition
P47S	Ch07_PTM, Ch11_IDR_Pro	[S/T]-P motif: S46 phosphorylation reduced; apoptosis impaired
**D49H**	Ch10_SLiM (Gate C)	TAD2 partner face: charge −1→+0.1 disrupts coupled folding with p300/NCBD
**D49N**	Ch10_SLiM (Gate C)	TAD2 partner face: charge −1→0 disrupts coupled folding
**I50T**	Ch10_SLiM (Gate C)	TAD2 partner face: β-branch loss + polar intro disrupts hydrophobic anchor
R290C	Ch07_PTM	±2 to PTM [K291/K292]: charge loss + reactive thiol
K292R	Ch07_PTM	Direct ubiquitin site: K → R loses ε-amino for isopeptide bond
G293W	Ch07_PTM, Ch12_IDR_Gly	±2 to PTM [K291/K292]: + 168 Å^3^ steric block + Gly freedom loss
S315T	Ch07_PTM	Direct phosphosite: S → T alters kinase substrate specificity
G374R	Ch07_PTM, Ch10_SLiM, Ch12_IDR_Gly	±2 to PTM [K372/K373]: charge intro + NLS2 + Gly freedom loss
Q375K	Ch10_SLiM	CT_reg: charge introduction in regulatory tail
R379S	Ch10_SLiM	NLS3: R → S charge loss abolishes nuclear import signal
R379L	Ch10_SLiM	NLS3: R → L charge loss + hydrophobic at signal position
R379H	Ch10_SLiM	NLS3 (outside Geta proximity): charge +1→+0.1, partial signal loss
E388A	Ch10_SLiM	CT_reg: E → A charge loss + desolvation
E388Q	Ch10_SLiM	CT_reg: E → Q charge loss; H-bond preserved, charge lost
D393Y	Ch07_PTM, Ch10_SLiM	±1 to PTM [S392]: charge loss + 82.5 Å^3^ volume gain

A separate observation supported the design of the Geta layer. R290H, classified as a True Molecular Positive in v17 (R290 is at ±1 to the K291/K292 ubiquitin sites; R → H reduces partner charge recognition), is no longer present in the v18 FINAL IDR FP list. This is not an indication of lost mechanism; it is the signature of Geta_IDR_PTM operating as designed. R ↔ H in IDR within ±1 of a PTM site is one of the three charge-preserving Geta exceptions (along with K ↔ R and S ↔ T), consistent with the observation that disordered backbones absorb the geometric perturbation while the partial charge of histidine retains substantial recognition by the E3 ligase. The framework therefore continues to flag charge-loss variants at the same position (R290C, −1→0, no Geta rescue) as TMP, while correctly releasing R290H. R290H thus illustrates the Geta layer doing precisely what the architecture intends: removing a specific subset of closures whose physical condition predicts tolerance, without lowering any threshold or weakening the underlying gate.

The remaining 17 IDR discordant variants did not yet have a sufficiently convincing physical rationale under v18 FINAL and were therefore retained as likely genuine false positives (Table 10). Importantly, these residual closures cluster in specific gate classes and specific molecular contexts, and each cluster names a specific gate that has not yet been written. The four new entries introduced in v18 (P4L, P4Q, D48G, P58A) all sit at or near the TAD1/TAD2 partner-face boundary and all involve directions of substitution that are physically tolerated but currently meet the Gate C non-conservative filter. P4 in particular illustrates this directly: P4R, a charge-introduction substitution at the same position, is correctly captured by Gate C as Pathogenic, while P4L (hydrophobic introduction) and P4Q (polar introduction with no charge change) are both ClinVar-Benign yet currently CLOSED. The three-variant pattern at P4 specifies a direction-aware filter (charge introduction CLOSED, hydrophobic / polar introduction OPEN at TAD-boundary positions) whose IF-THEN form is established side-chain chemistry; the corresponding Geta is scheduled for the next package release, with the manuscript evaluation held fixed at v0.5.1 (v18 FINAL) for reproducibility. D48G similarly corresponds to a position outside the current 6-partner union (positions 48 and 57 are not present in the 59-residue union face); incorporation of additional partner complexes covering these positions would resolve this case. The carry-over Genuine FPs from v17 (in PRD and CTD; involving Ch10_SLiM, Ch11_IDR_Pro, Ch12_IDR_Gly) similarly point to specific sub-gate refinements rather than threshold retuning.

When the 19 mechanistically supported discordances were reinterpreted as true molecular positives, the apparent performance of the framework improved substantially without changing the primary evaluation definition. Under this molecularly adjusted analysis, sensitivity rose from 84.5% to 85.0%, specificity from 50.0% to 58.3%, and PPV from 89.1% to 92.2% (Table 11). We emphasize that this adjusted analysis does not replace the primary ClinVar-based evaluation. Rather, it provides an orthogonal test of the central claim of the framework: Gate & Channel operates at the level of explicit molecular mechanism, whereas clinical databases necessarily aggregate penetrance, ancestry, ascertainment, and disease-definition effects.

**Table 11:** Standard versus molecularly adjusted performance (v18 FINAL). The adjusted analysis reclassifies the 19 IDR variants with explicit physical disruption rationales (Table 9) from FP to TP.

Metric	Standard	Mol-adjusted
TP	547	566
FP	67	48
FN	100	100
TN	67	67
Sensitivity	84.5%	85.0%
Specificity	50.0%	58.3%
PPV	89.1%	92.2%

**Fig 4:**
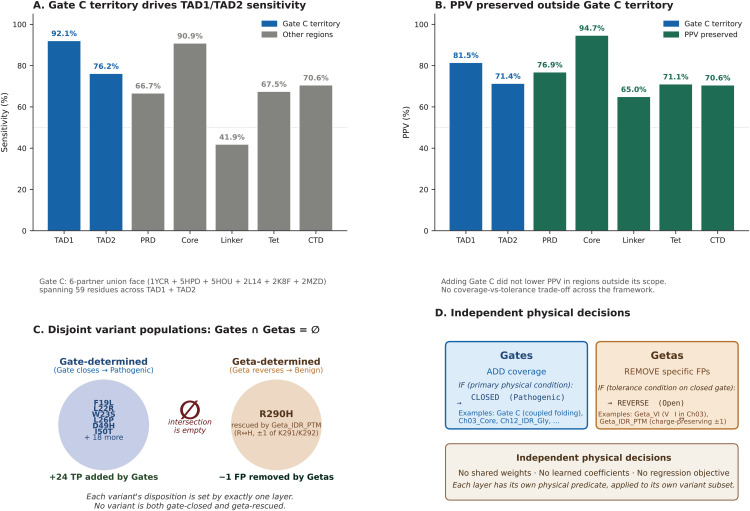
Gate–Geta disjoint operation, evidenced across full-length TP53. A: Per-region sensitivity in v18 FINAL. Gate C territory (TAD1, TAD2; blue) reaches 92.1% and 76.2% respectively; other regions (gray) show their independent baselines (Core 90.9%, PRD 66.7%, Tet 67.5%, CTD 70.6%, linker 41.9%). B: Per-region positive predictive value in v18 FINAL. Adding the multi-partner Gate C did not lower PPV in regions outside its scope, indicating that the framework gains coverage in TAD1/TAD2 without imposing a tolerance penalty elsewhere — there is no global coverage–specificity trade-off. C: Disjoint variant populations. The set of variants whose disposition is determined by Gates (left, blue: representative TAD1/TAD2 variants F19L, L22R, W23S, L26P, D49H, I50T, plus 18 others) and the set of variants whose disposition is determined by Getas (right, amber: R290H, rescued by Geta_IDR_PTM via the R ↔ H, ±1 to K291/K292 rule) do not overlap; their intersection is empty. Each variant’s disposition is set by exactly one layer. D: Concept summary. Gates ADD coverage by closing on a primary physical condition; Getas REMOVE specific false positives by reversing closure under a secondary tolerance condition. The two layers share no weights, no learned coefficients, and no regression objective; each layer applies its own physical predicate to its own variant subset.

Overall, these results support a key distinction underlying the framework. Gate & Channel is not optimized to mimic clinical labels or to maximize a fitted performance objective. Instead, it is designed to identify discrete physical mechanism failure. Where that mechanistic layer coincides with clinical pathogenicity, the framework behaves as a conventional classifier. Where it diverges, especially in IDRs, the discordance itself becomes informative, separating cases where a specific gate has not yet been written from cases of molecular–clinical decoupling. The full per-region picture, together with the disjoint variant populations on which Gates and Getas operate, is summarized in Fig 4.

### The same channels carry meaning beyond TP53: cross-protein validation on KRAS, TDP-43, and BRCA1

**Fig 5:**
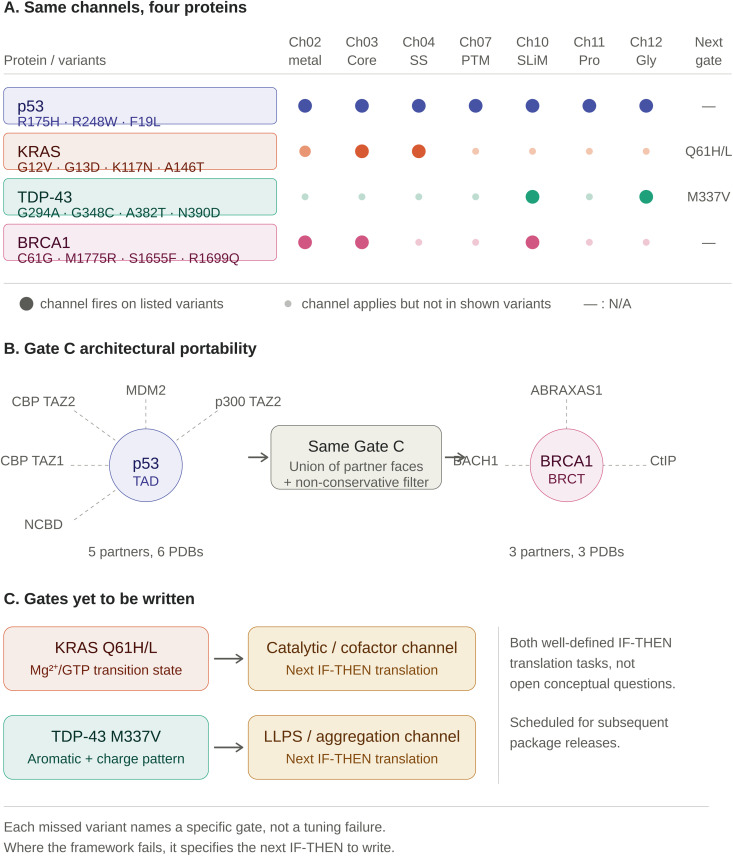
Cross-protein validation: the same channels capture pathogenic mechanisms in KRAS, TDP-43, and BRCA1. A: Schematic of the four bundled proteins and the channels that fire on representative pathogenic variants in each. The same channels (Ch02 metal coordination, Ch03 buried packing, Ch04 secondary structure, Ch10 SLiM / Gate C, Ch11 IDR proline, Ch12 IDR glycine, Ch07 PTM logic) operate without modification across all four proteins. B: Architectural portability of Gate **C.** The “union of partner faces + non-conservative filter” design used for p53 (MDM2 + CBP TAZ2/TAZ1/NCBD + p300 TAZ2; PDBs 1YCR, 5HPD, 5HOU, 2L14, 2K8F, 2MZD) transfers 1: 1 to BRCA1 BRCT (ABRAXAS1 + BACH1 + CtIP; PDBs 1T29, 1T15, 1Y98), with no logic change. C: Variants whose physical mechanism falls outside any current channel name a specific gate yet to be written: KRAS Q61H/L → catalytic / cofactor channel; TDP-43 M337V → aggregation / LLPS channel.

The most direct way to ask whether the channels developed for TP53 carry physical meaning beyond TP53 is to apply them, without modification, to other proteins and to record for each variant which p53-derived channel captures it and which channel would need to be added. We did this for three additional cancer-relevant proteins — KRAS (a small GTPase oncogene), TDP-43 (an RNA-binding protein implicated in ALS), and BRCA1 (a tumor-suppressor with RING and BRCT domains) — using the same pathogenicity-gates CLI used for the TP53 evaluation. No protein-specific code path was required; each protein was added as a single YAML annotation file plus a PDB or AlphaFold structure. Per-variant predictions and channel firing patterns are provided in S6 Table; the high-level per-protein outcome is shown in Fig 5.

The per-protein outcome is summarized in Table 12. For each protein, p53-derived channels capture the dominant pathogenic mechanism, and the residual missed variants name a specific physical regime whose IF-THEN translation has not yet been written.

**Table 12:** Cross-protein validation: variants captured by p53-derived channels and gates yet to be written. For each protein, the variants captured by the existing 12 channels and 2 Getas are listed together with the channel responsible. Variants for which no current channel encodes the relevant physics name a specific gate scheduled for subsequent package releases.

Protein	Variants captured by p53-derived channels	Gate yet to be written
KRAS	G12V/D/C, G13D, K117N, A146T (Ch03_Core, Ch04_SS); K184Q (HVR; UNIVERSAL IDR charge logic)	GTP-cofactor channel for Q61H/L (transition-state geometry around Mg^2 +^ /GTP)
TDP-43	G294A, G295V, G348C (Ch12_IDR_Gly); A315T, A382T (Ch10 PPII spacer β-branch); N382D, N390D (Ch10 IDR charge introduction)	Aggregation / LLPS channel for M337V (aromatic spacing, charge patterning)
BRCA1 (RING)	C61G, C64R, T37R (Ch02 Zn-analog; Ch03 charge)	—
BRCA1 (BRCT, buried)	V1696L, K1702N, A1708E, M1775R, C1697R (Ch03_Core)	—
BRCA1 (BRCT, phospho-pocket)	S1655F, R1699Q, R1699W, S1715R (Gate C with partners ABRAXAS1, BACH1, CtIP; PDBs 1T29, 1T15, 1Y98)	— (within tested set)

Three observations follow directly. *First*, the p53 channels carry meaning for other proteins. The same physical machinery — buried-site integrity (Ch03_Core), secondary-structure strain (Ch04_SS), Zn-coordination logic (Ch02_Zn applied as a generalized metal-binding gate), surface and IDR charge logic, IDR backbone constraints (Ch11_IDR_Pro, Ch12_IDR_Gly), and Gate C (the multi-partner coupled-folding face) — activates on the corresponding mechanism in each of the four bundled proteins, without requiring any protein-specific code path. *Second*, Gate C is architecturally portable: replacing the p53 partner set {MDM2, CBP TAZ2/TAZ1/NCBD, p300 TAZ2} with the BRCA1 BRCT partner set {ABRAXAS1, BACH1, CtIP} produces a working Gate C for the BRCT phospho-pocket variants without modifying the channel logic itself. The “union of partner faces + non-conservative filter” pattern is protein-independent. *Third*, where a channel is missing, the framework names what to add: KRAS Q61H/L specifies a catalytic / cofactor channel based on transition-state geometry around Mg^2+^/GTP, and TDP-43 M337V specifies an aggregation / LLPS channel based on aromatic spacing and charge patterning. Both are well-defined IF-THEN translation tasks rather than open conceptual questions, and both are scheduled for subsequent package releases.

The same logic applies to the cross-protein case: each missing channel is a specific physical input — a partner-complex structure, a cofactor geometry, a phase-separation motif — whose IF-THEN translation completes the loop. The transferability classification of the 12 channels and 2 Getas (UNIVERSAL: applicable from sequence and annotation alone; ADAPTABLE: applicable from coordinates, including AlphaFold predictions with pLDDT > 70; SPECIFIC: requires target-specific structural input) is summarized in S5 Table.

## Discussion

In this study, we developed a zero-parameter, first-principles Gate & Channel framework for full-length TP53 missense variant analysis and showed that explicit, auditable IF-THEN gates and post-closure exception rules (Getas) can recover substantial pathogenic signal across both structured and intrinsically disordered regions. In the primary ClinVar-based evaluation, the v18 FINAL framework achieved 84.5% sensitivity and 89.1% positive predictive value while preserving 90.9% sensitivity in the core DNA-binding domain and maintaining complete capture of the nine canonical hotspot mutations. These results are notable not because the framework was optimized to maximize benchmark performance, but because it was deliberately not optimized in that way. No thresholds were fitted to ClinVar labels, no channel weights were learned, and no latent score was trained to imitate clinical classifications. Instead, the framework was built from discrete physical constraints: DNA-contact geometry, Zn coordination, burial-dependent packing logic, secondary-structure compatibility, interface perturbation, PTM chemistry, SLiM logic, and IDR backbone constraints. The central result is therefore not simply that TP53 can be classified with useful accuracy, but that much of this classification can be written explicitly as mechanism, and that the same channels carry meaning when applied to other proteins (KRAS, TDP-43, BRCA1) in representative test cases.

This distinction matters because most variant-interpretation frameworks are judged primarily by aggregate predictive metrics, even when their internal reasoning is difficult to audit. Gate & Channel was designed under a different objective. A prediction is not the output of a fitted score, but the consequence of one or more closed gates, each corresponding to a specific physical violation. This architecture makes each call decomposable. A variant may be predicted disruptive because it abolishes a direct DNA contact, destabilizes the Zn environment, creates a buried cavity, introduces a buried steric clash, destroys a proline-directed kinase motif, disrupts an import signal, or removes the backbone freedom required in a disordered segment. Such decomposition does not merely improve interpretability in a cosmetic sense; it changes what the model is. Rather than a black-box predictor that happens to correlate with pathogenicity, the framework functions as a mechanistic audit system for missense substitutions.

A key result of the present work is that this logic can be extended beyond the ordered p53 core into the full-length protein without sacrificing structured-domain performance. This was not guaranteed. Full-length TP53 spans fundamentally different physical regimes: a folded DNA-binding core, an oligomeric tetramerization domain, and multiple intrinsically disordered regions whose function depends on coupled folding, sequence motifs, PTM access, localization signals, and polymer-like backbone behavior. One possible failure mode of full-length expansion would have been that adding disorder-specific rules degraded performance in the canonical structured regions. Instead, core-domain sensitivity was preserved, suggesting that the framework extension was additive rather than destabilizing. Methodologically, this supports the design choice of treating ordered and disordered contexts through separate but compatible gate families, rather than forcing them into a single feature space or continuous score.

The IDR results are particularly important conceptually. Intrinsically disordered regions are often treated as difficult cases for mechanistic interpretation because they lack a stable tertiary structure that can be interrogated by conventional structural predictors. Here, however, useful signal was recovered through one-dimensional gates rooted in explicit physical logic. The Ch10 SLiM gates captured coupled folding at the MDM2-binding face, polyproline-II incompatibility in the proline-rich domain, spacer β-branch disruption, NLS charge loss, aromatic anchor destruction, and charge-pattern perturbation in the C-terminal tail. The PTM channel recovered both direct chemical hits and local perturbations of PTM accessibility, while the IDR-specific proline and glycine gates formalized two complementary backbone principles: local proline-dependent folding constraints and the functional importance of glycine-mediated conformational freedom. These results argue against the notion that disorder is merely an absence of structure that must be compensated by statistical learning. Rather, disorder appears here as a regime governed by local, context-dependent constraints that can themselves be formalized mechanistically.

Several of the new IDR gates are also biologically informative in their own right. The proline-directed kinase gate provides one example. At validated [S/T]-P phosphomotifs, the framework captured all curated pathogenic Pro → X substitutions, supporting the interpretation that the proline ring itself acts as part of the kinase recognition element rather than as a generic hydrophobic residue. Likewise, the SLiM-boundary N-cap gate at P12 suggests that proline can act as a folding delimiter at the edge of a coupled-folding region, with loss of that delimiter potentially permitting inappropriate helix propagation. Ch12_IDR_Gly, the IDR glycine constraint, introduces a related but distinct idea: in a disordered region, the absence of a side chain may be required for function. This is a useful conceptual shift. In folded domains, function is often associated with the presence of a precisely packed structure. In disordered regions, function may instead depend on preserved conformational latitude, and substitutions that reduce that latitude can therefore be mechanistically disruptive even in the absence of a stable tertiary fold.

The discordant IDR calls further clarify what the framework is and is not attempting to measure. In the standard ClinVar evaluation, some variants classified as Benign were predicted disruptive by one or more IDR gates, contributing to the modest overall specificity. However, a post hoc audit showed that many of these apparent false positives had explicit physical rationales consistent with true molecular disruption, including PTM-proximal steric or electrostatic perturbation, direct modification-site chemistry changes, loss of NLS basicity, and disruption of the S46-P47 phosphoregulatory module. The P47S example is particularly instructive: although ClinVar classifies the variant as benign, biochemical studies have shown reduced S46 phosphorylation and impaired apoptotic response [17]. In the language of the present framework, this is not an error but a resolution mismatch. Gate & Channel operates at the level of molecular mechanism disruption, whereas clinical databases necessarily collapse molecular function, penetrance, ancestry, ascertainment, and disease-definition effects into a single label. For this reason, the molecularly adjusted analysis should not replace the primary ClinVar evaluation, but it does support the view that some discordant calls in disordered regions reflect molecular–clinical decoupling rather than incorrect physics.

Equally important, the remaining discordances function as a map of next gates to be written. In a gate-based framework, missed variants are not invitations to lower thresholds; they specify the physical mechanism whose IF-THEN translation has not yet been written.

### Next gates to be written

Within TP53, the v18 FINAL error pattern points to several specific gates whose implementation is a finite physical translation rather than an open conceptual question. Five categories were identified within p53 itself (conservative substitutions in partner-specific pockets that have not yet been incorporated into the union face; non-proline positions in the proline-rich domain that interact with context-specific binding partners; linker variants between the core and tetramerization domains where binding or dynamics information is required; the 360–362 boundary at the start of the C-terminal tail; and isolated IDR prolines whose function depends on polymer-level persistence or compaction). At TAD1 position 4, the contrast between P4R (Pathogenic, charge introduction; correctly captured by Gate C) and P4L / P4Q (both Benign; closed by Gate C as false positives) specifies a direction-aware filter whose IF-THEN form is immediate: charge introduction CLOSED, hydrophobic / polar introduction OPEN at TAD-boundary positions. The cross-protein evaluation extends the same map: KRAS Q61H/L specifies a catalytic / cofactor channel based on transition-state geometry around Mg^2+^/GTP; TDP-43 M337V specifies an aggregation / LLPS channel based on aromatic spacing and charge patterning; the framework’s lack of an exclusion channel (the inverse of Gate C) is well-defined as the absence of an interaction whose presence would have been disruptive.

In each of these cases, the physical input is independently studied; what stands between the current framework and the captured variant is a finite IF-THEN translation. The corresponding gates are scheduled for subsequent releases of the package; the manuscript evaluation is held fixed at v0.5.1 (v18 FINAL) for reproducibility, not because their construction is open-ended. In this sense, the framework does not only classify variants; it organizes the queue of physics to be written.

The present study also highlights a deliberate tradeoff between mechanistic explicitness and benchmark optimization. Because thresholds were not fitted to labels and because the decision rule was binary rather than weighted, the framework is not expected to dominate purely statistical methods on every aggregate metric. This is especially true for specificity, where clinical labeling and molecular mechanism can diverge. However, the framework gains something that fitted predictors generally lack: the ability to state why a variant was called, which physical assumption was invoked, and which missing mechanisms are implicated when the call fails. That tradeoff is not incidental; it is the central methodological choice of the study. Indeed, the binary rule *n*_closed_ ≥ 1 may appear severe from a statistical perspective, but it reflects a mechanistic claim: a single indispensable physical constraint can be sufficient for molecular failure. Requiring agreement among multiple gates would improve conservatism at the cost of obscuring sparse but decisive lesions such as direct coordination-site disruption or motif-destroying substitutions.

### Scope of v18 FINAL

It is useful to be explicit about what v18 FINAL does and does not address. First, the framework’s coverage is determined by the physical mechanisms that have been encoded as IF-THEN gates so far; regions of TP53 (or of any other protein) for which structural or biochemical annotation is sparse are correspondingly under-represented in the current channel set. Importantly, this is not a fundamental property of the framework but a state of its current implementation: each missing region names a specific gate whose physical input is identified above. Second, although thresholds were not fitted to ClinVar, they are instantiated in TP53-specific structural contexts; for ordered-domain channels they are expected to transfer to AlphaFold-derived structures with pLDDT > 70 (Methods), but threshold values themselves should be re-confirmed when the framework is applied to a substantially different structural context. Third, the present evaluation is anchored to ClinVar categories, which are imperfect surrogates for molecular function; the molecularly adjusted analysis (Section 3.3) is intended to make this distinction explicit, not to replace the primary evaluation. Fourth, the framework is not intended for clinical deployment without additional validation, particularly in cases where molecular disruption and disease penetrance can diverge. Finally, the current decision rule is single-variant and local; epistasis, isoform dependence, cell-state dependence, and higher-order regulatory effects are not captured by this version.

Each of these scopes points to a specific construction. AlphaFold-driven extension to additional proteins is implemented in the bundled CLI; partner-specific Gate C extensions are implementable as YAML annotation additions; epistatic and combinatorial extensions require a multi-variant generalization of the decision rule but no fundamental architectural change.

The Gate & Channel formalism, taken as a whole, suggests a broader strategy for interpretable computational biology. Its building blocks are discrete, explicit, auditable, and symmetry-aware. They can be inspected, criticized, revised, and extended one mechanism at a time. This makes the framework not only compatible with human mechanistic reasoning, but also potentially compatible with future agent-based scientific workflows in which computational systems propose, test, and refine candidate gates under strict first-principles constraints. The present study does not claim that TP53 has been fully solved; it claims, more narrowly and more concretely, that a substantial fraction of TP53 missense variation can be understood without regression, without hidden weights, and without surrendering mechanism to black-box fitting — and that the same gates carry meaning beyond TP53. In that sense, the main contribution of this work is both practical and conceptual: it provides a full-length, cross-protein-tested mechanistic analysis of TP53 and establishes a computational language in which variant interpretation can be expressed as explicit physical law-like rules rather than as opaque statistical resemblance, with each remaining failure naming the next rule to be written.

## Conclusion

We developed a zero-parameter, first-principles Gate & Channel framework for full-length TP53 missense variant analysis and showed that a substantial fraction of variant effects can be resolved through explicit, auditable IF-THEN gates and post-closure exception rules (“Getas”) rather than fitted scores alone. By combining structured-domain channels with IDR-specific channels and a hierarchical Gate–Geta layer, the framework preserved strong performance in the canonical DNA-binding core while extending mechanistic coverage to disordered regions that are often difficult to analyze by conventional structure-centered methods. We further showed that the same channels carry meaning beyond TP53: applied to KRAS, TDP-43, and BRCA1, they capture the dominant pathogenic mechanism in each protein and, where they fail, name a specific gate yet to be written. In this formulation, a prediction is not merely a label, but a statement about which physical constraint is violated; and a missed variant is not a tuning failure, but a specification of what physics to translate next.

We do not view this framework as an argument against regression- or machine-learning-based approaches. Fitted models can capture statistical signal that explicit physical gates do not yet encode, and such approaches will remain valuable for variant interpretation. Instead, we see these strategies as complementary. Regression-based methods can be powerful in detecting complex patterns in data, whereas Gate & Channel provides a parallel framework in which variant effects can be explained, audited, and revised one physical mechanism at a time.

In that sense, Gate & Channel does not claim novelty by departing from prior knowledge, but by relying on it deliberately. The framework is built by standing on established structural biology, physicochemistry, geometry, and polymer physics, and by turning those accumulated observations into explicit rules that can be tested, criticized, and extended. The corresponding implementation is distributed as the open-source Python package pathogenicity-gates (v0.5.1, MIT license, pip install pathogenicity-gates), with a command-line interface and bundled annotations for p53, KRAS, TDP-43, and BRCA1; adding a new target protein requires only a YAML annotation file and a PDB or AlphaFold structure. We hope that this kind of mechanistic formalization will be useful in clinical interpretation, where transparent rationale may matter alongside aggregate predictive performance. More broadly, we hope that future AI systems will advance not only regression-based predictors but also auditable first-principles frameworks, by helping identify physical mechanisms whose IF-THEN translation has not yet been written and proposing new gates that remain interpretable to human investigators.

## Supporting information

S1 Text Extended Gate & Channel logic for full-length TP53 missense variant interpretation. Complete gate specifications for all 12 channels and 2 Getas, including structured-domain channels (Ch01–Ch09), IDR-specific channels (Ch10_SLiM, Ch11_IDR_Pro, Ch12_IDR_Gly), the IDR sub-gates of Ch07_PTM, the post-closure exception layer (Geta_VI, Geta_IDR_PTM), symmetry verification, tier classification, and the distinction between molecular mechanism disruption and clinical penetrance.
(PDF)

S1 Table Variant-level predictions for all in-scope TP53 missense variants. Complete classification results for all 1,369 in-scope variants with ClinVar label, prediction, number of closed gates, and specific channels that returned CLOSED.
(XLSX)

S2 Table Audit of IDR-discordant variants. Mechanistic audit of the 36 IDR variants classified as Benign in ClinVar but predicted disruptive in v18 FINAL, with assignment to True Molecular Positive (19) or Genuine False Positive (17) categories and physical rationales.
(XLSX)

S3 Table Channel- and gate-level firing statistics. Firing counts for each channel and gate across all variants, broken down by ClinVar classification.
(XLSX)

S4 Table False-negative categorization and map of next gates to be written. Categorization of IDR false negatives into five mechanistic classes, with physical explanations and data needed to resolve each category.
(XLSX)

S5 Table Transferability matrix. Classification of all 12 channels and 2 Getas as UNIVERSAL (applicable from sequence and annotation alone), ADAPTABLE (applicable from coordinates, including AlphaFold predictions with pLDDT > 70), or SPECIFIC (requires target-specific structural input). Includes per-channel notes on AlphaFold compatibility and on architectural portability of Gate C. Provided as.
(XLSX)

S6 Table Cross-protein gate firing. Per-variant gate firing patterns for KRAS, TDP-43, and BRCA1 used in the cross-protein validation (Section 3.4 and Fig 5). Each row contains the variant, the channel(s) closed, and the corresponding p53-derived physical mechanism.
(XLSX)

S2 Text Computational implementation and reproducibility details. Repository structure, CLI usage (pathogenicity-gates predict, predict-batch, explain, list-proteins), input file provenance, YAML annotation schema for adding new target proteins, evaluation logic, and reproducibility notes.
(PDF)

S1 Dataset. **Repository manifest.** Complete manifest of the GitHub repository, including bundled YAML annotations and reference structures for p53, KRAS, TDP-43, and BRCA1.
(PDF)

### Use of AI tools

The author is a non-native English speaker. Manuscript preparation involved structured drafting in Japanese followed by translation to academic English with the assistance of Anthropic Claude Opus 4.6. AI translation assistance was applied to (i) Japanese-to-English conversion of section drafts, (ii) academic phrasing and stylistic refinement, and (iii) editorial consistency checks across the manuscript. All translated text was reviewed by the author against the original Japanese drafts and against the cited literature. Implementation support for the open-source pathogenicity-gates Python package — including code structuring, command-line interface design, test scaffolding, and documentation — was performed with the assistance of Claude Code (Anthropic). All gate definitions, channel logic, physical reasoning, threshold derivations, benchmark evaluations, and scientific interpretations were conceived and validated by the human author. AI-assisted code was verified by direct execution against the bundled ClinVar dataset and by per-variant audit of channel firing patterns reported in the manuscript. No AI tool was used as an author or as a substitute for scientific judgment, and no AI tool was used to fabricate or modify primary research data.

## References

[1] Joerger AC, Fersht AR. The tumor suppressor p53: from structures to drug discovery. Cold Spring Harb Perspect Biol. 2010;2(6):a000919. doi: 10.1101/cshperspect.a000919 20516128 PMC2869527

[2] Wright PE, Dyson HJ. Intrinsically disordered proteins in cellular signalling and regulation. Nat Rev Mol Cell Biol. 2015;16(1):18–29. doi: 10.1038/nrm3920 25531225 PMC4405151

[3] Oldfield CJ, Dunker AK. Intrinsically disordered proteins and intrinsically disordered protein regions. Annu Rev Biochem. 2014;83:553–84. doi: 10.1146/annurev-biochem-072711-164947 24606139

[4] Ng PC, Henikoff S. SIFT: Predicting amino acid changes that affect protein function. Nucleic Acids Res. 2003;31(13):3812–4. doi: 10.1093/nar/gkg509 12824425 PMC168916

[5] Adzhubei IA, Schmidt S, Peshkin L, Ramensky VE, Gerasimova A, Bork P, et al. A method and server for predicting damaging missense mutations. Nat Methods. 2010;7(4):248–9. doi: 10.1038/nmeth0410-248 20354512 PMC2855889

[6] Cheng J, Novati G, Pan J, Bycroft C, Žemgulytė A, Applebaum T, et al. Accurate proteome-wide missense variant effect prediction with AlphaMissense. Science. 2023;381(6664):eadg7492. doi: 10.1126/science.adg7492 37733863

[7] Kircher M, Witten DM, Jain P, O’Roak BJ, Cooper GM, Shendure J. A general framework for estimating the relative pathogenicity of human genetic variants. Nat Genet. 2014;46(3):310–5. doi: 10.1038/ng.2892 24487276 PMC3992975

[8] Ioannidis NM, Rothstein JH, Pejaver V, Middha S, McDonnell SK, Baheti S, et al. REVEL: An Ensemble Method for Predicting the Pathogenicity of Rare Missense Variants. Am J Hum Genet. 2016;99(4):877–85. doi: 10.1016/j.ajhg.2016.08.016 27666373 PMC5065685

[9] Kussie PH, Gorina S, Marechal V, Elenbaas B, Moreau J, Levine AJ, et al. Structure of the MDM2 oncoprotein bound to the p53 tumor suppressor transactivation domain. Science. 1996;274(5289):948–53. doi: 10.1126/science.274.5289.948 8875929

[10] Sims JJ, Scavone F, Cooper EM, Kane LA, Todi SV, Bhatt D, et al. Phosphorylation site analysis in signal transduction. Methods Mol Biol. 2013;1002:1–23.23625390

[11] Landrum MJ, Lee JM, Benson M, Brown GR, Chao C, Chitipiralla S, et al. ClinVar: improving access to variant interpretations and supporting evidence. Nucleic Acids Res. 2018;46(D1):D1062–7. doi: 10.1093/nar/gkx1153 29165669 PMC5753237

[12] Cho Y, Gorina S, Jeffrey PD, Pavletich NP. Crystal structure of a p53 tumor suppressor-DNA complex: understanding tumorigenic mutations. Science. 1994;265(5170):346–55. doi: 10.1126/science.8023157 8023157

[13] Malecka KA, Ho WC, Bhakta SK, Olanich ME, Hubbard R, Cowan N, et al. Crystal structure of human p53 tetramerization domain. Protein Data Bank entry 2J0Z. 2008.

[14] The UniProt Consortium. UniProt: the Universal Protein Knowledgebase in 2025. Nucleic Acids Res. 2025;53(D1):D294–9. 10.1093/nar/gkae1010 PMC11701636 39552041

[15] van der Lee R, Buljan M, Lang B, Weatheritt RJ, Daughdrill GW, Dunker AK, et al. Classification of intrinsically disordered regions and proteins. Chem Rev. 2014;114(13):6589–631. doi: 10.1021/cr400525m 24773235 PMC4095912

[16] Kumar M, Gouw M, Michael S, Sámano-Sánchez H, Pancsa R, Glavina J, et al. ELM-the eukaryotic linear motif resource in 2020. Nucleic Acids Res. 2020;48(D1):D296–306. doi: 10.1093/nar/gkz1030 31680160 PMC7145657

[17] Whibley C, Pharoah PDP, Hollstein M. p53 polymorphisms: cancer implications. Nat Rev Cancer. 2009;9(2):95–107. doi: 10.1038/nrc2584 19165225

[18] Adzhubei AA, Sternberg MJE, Makarov AA. Polyproline-II helix in proteins: structure and function. J Mol Biol. 2013;425(12):2100–32. doi: 10.1016/j.jmb.2013.03.018 23507311

[19] Liang SH, Clarke MF. Regulation of p53 localization. Eur J Biochem. 2001;268(10):2779–83. doi: 10.1046/j.1432-1327.2001.02227.x 11358492

[20] Jumper J, Evans R, Pritzel A, Green T, Figurnov M, Ronneberger O, et al. Highly accurate protein structure prediction with AlphaFold. Nature. 2021;596(7873):583–9. doi: 10.1038/s41586-021-03819-2 34265844 PMC8371605

[21] Evans R, O’Neill M, Pritzel A, Antropova N, Senior A, Green T, et al. Protein complex prediction with AlphaFold-Multimer. bioRxiv. 2022. doi: 10.1101/2021.10.04.463034

[22] Bouaoun L, Sonkin D, Ardin M, Hollstein M, Byrnes G, Zavadil J, et al. TP53 Variations in Human Cancers: New Lessons from the IARC TP53 Database and Genomics Data. Hum Mutat. 2016;37(9):865–76. doi: 10.1002/humu.23035 27328919

