# Supplementary material for: A zero-parameter first-principles gate framework for full-length TP53 missense variant interpretation
Source: PLoS Comput Biol. 2026 Jun 11;22(6):e1014168. doi: 10.1371/journal.pcbi.1014168 (PMC13278589; doi:10.1371/journal.pcbi.1014168)
Supplement: S1 Text — Complete gate specifications for all 12 channels and 2 Getas, including structured-domain channels (Ch01–Ch09), IDR-specific channels (Ch10_SLiM, Ch11_IDR_Pro, Ch12_IDR_Gly), the IDR sub-gates of Ch07_PTM, the post-closure exception layer (Geta_VI, Geta_IDR_PTM), symmetry verification, tier classification, and the distinction between molecular mechanism disruption and clinical penetrance. (PDF) [file pcbi.1014168.s001.pdf]

S1 Text

Extended Gate & Channel logic for full-length TP53 missense variant interpretation

For: A zero-parameter first-principles gate framework for full-length TP53 missense variant interpretation

Author: Masamichi Iizumi

S1.1 Framework overview

Gate & Channel (v18 FINAL) is a zero-parameter, first-principles framework for full-length TP53 missense variant interpretation. Each missense variant is evaluated through 12 independent Channels (Ch01–Ch12), each containing one or more binary Gates that test a specific physical constraint. When the constraint is disrupted by the substitution, the Gate returns CLOSED; a variant is predicted disruptive if any Gate across any Channel closes ($n_{\mathrm{closed}} \geq 1$). A second hierarchical layer of two Getas (Geta_VI for V↔I exchange in buried β-branched positions; Geta_IDR_PTM for charge-preserving substitutions adjacent to PTM sites in IDR) reverses specific closures whose physical condition predicts tolerance. No channel weights are learned, no continuous pathogenicity score is fitted, and thresholds are not tuned against ClinVar labels. The framework was designed under four principles: (i) thresholds are derived from physical interpretation rather than label fitting; (ii) rules must be expressible as explicit IF–THEN gates; (iii) missed pathogenic variants are interpreted as evidence that a specific physical mechanism has not yet been encoded as a gate, rather than evidence that thresholds are too strict; and (iv) when a channel expansion produces incidental closures on legitimately tolerated variants, a physically grounded post-closure exception (Geta) is added rather than the underlying gate weakened.

S1.2 Input data and modeled scope

The ordered DNA-binding core domain was modeled from 1TSR, using chain B for the protein and DNA chains E/F for side-chain-to-DNA distance calculations. The tetramerization domain was modeled from 2J0Z. The coupled-folding interface between the N-terminal transactivation domain and partner proteins was defined from a 6-partner union of complex structures (1YCR for MDM2; 5HPD and 2K8F for CBP TAZ2; 5HOU for CBP TAZ1; 2L14 for p300 TAZ2; 2MZD for NCBD), yielding a 59-residue union face spanning TAD1 and TAD2. Post-translational modification annotations were taken from UniProt P04637. The full-length framework covers the core domain (residues 94–289), the tetramerization domain (325–356), and intrinsically disordered regions in the N terminus (1–93), linker (290–324), and C-terminal tail (357–393). Structured regions are analyzed with three-dimensional gates derived from coordinates, whereas IDR

segments are analyzed with one-dimensional gates derived from motif context, PTM proximity, charge logic, and backbone freedom constraints.

S1.3 Structured-domain channels

Ch01_DNA encodes DNA-contact disruption using side-chain heavy-atom distances to DNA and physicochemical changes in charge and hydrogen-bonding capacity. **Ch02_Zn** encodes Zn-coordination logic through a layered cascade, including direct ligand disruption and electrostatic perturbation of the Zn environment. **Ch03_Core** encodes symmetry-complete core integrity logic, including buried cavity formation, steric clash, hydrogen-bond loss or gain, hydrophobicity reversal, sulfur-rich environments, β-branching constraints, surface hydrophobic exposure, and electrostatic keystone networks. **Geta_VI** acts on Ch03_Core as a post-closure exception for V$\leftrightarrow$I substitutions in buried β-branched positions, where both rotamers are physically interchangeable. **Ch04_SS** tests local secondary-structure incompatibility in helices and β-strands. **Ch05_Loop** captures structured-region glycine and proline backbone constraints and loop-anchor sensitivity. **Ch06_PPI** models protein–protein interface perturbation from the union of 16 resolved p53 interaction structures (66-residue interface set in the core domain). **Ch07_PTM** encodes direct PTM-site chemistry and structured-region PTM proximity (OR logic for charge and volume perturbation within $\pm$2 residues), with a separate set of IDR sub-gates described below. **Ch08_Oligomer** (tetramer interface in p53) captures interface disruption through exposure asymmetry and rigid multi-chain hub logic. **Ch09_SaltBridge** models disruption of surface salt-bridge networks.

S1.4 IDR-specific channels

IDR regions were treated not as featureless sequence, but as segments whose function is encoded by local, context-dependent constraints. **Ch11_IDR_Pro** models Pro$\rightarrow$X events in a context-dependent manner, distinguishing polyproline-II structure, SLiM interior positions, SLiM boundary prolines, PTM-proximal prolines, and isolated IDR prolines; the known benign polymorphism P72 (rs1042522) is explicitly excluded. The IDR sub-gates of Ch07_PTM are split in a mutually exclusive and collectively exhaustive (MECE) manner from the structured-region PTM logic: direct PTM-site chemistry, structured-region proximity rules ($\pm$2, OR logic), IDR proximity rules ($\pm$1, OR logic), and proline-directed kinase [S/T]-P motifs. **Geta_IDR_PTM** acts on the IDR proximity sub-gates as a post-closure exception for charge-preserving swaps (K$\leftrightarrow$R, R$\leftrightarrow$H, S$\leftrightarrow$T) within $\pm$1 residue of a PTM site. **Ch10_SLiM** groups SLiM-level logic, including coupled folding (Gate C from the 6-partner union 1YCR/5HPD/5HOU/2L14/2K8F/2MZD; CLOSED requires both partner-face residence and a non-conservative substitution $|\Delta Q| \geq 0.3$ or $|\Delta V| \geq 30$ Å^3 or $|\Delta h| \geq 1.5$),

PPII incompatibility (aromatic introduction in PRD), PPII spacer β-branch disruption ($\{A,G\}\to\{V,I,T\}$ at spacer positions), nuclear localization signal charge loss (NLS1/NLS2/NLS3), aromatic anchor loss (W53, F54 in BOX_II), and C-terminal regulatory charge-pattern disruption. **Ch12_IDR_Gly** formalizes the IDR glycine constraint: in disordered regions, the absence of a side chain can itself be functional, and G→X substitutions are therefore treated as losses of backbone freedom.

S1.5 Symmetry, tiers, and decision logic

Where the underlying physics is bidirectional, Gate & Channel encodes symmetric perturbation pairs rather than one-sided heuristics. Verified mirror pairs include cavity versus steric clash, Gly→X versus X→Gly, Pro→X versus X→Pro, charge loss versus charge introduction, charge sign reversal (self-symmetric), hydrophobic-to-polar versus polar-to-hydrophobic substitutions, hydrogen-bond loss versus hydrogen-bond gain, aromatic loss versus aromatic gain, and β-branch loss versus steric equivalent. In the IDR, PPII compatible→incompatible (Gate A) and PPII spacer→ β-branch (Gate A2) represent additional verified pairs.

Gates are classified into three tiers. Tier S (Supreme) gates represent hard chemical or backbone constraints with zero observed benign variants in the modeled context. Tier A (Structural) gates capture burial- and network-dependent structural perturbations with 1–3 benign exceptions. Tier B (Functional) gates encode annotation-dependent chemistry such as PTM-site identity or motif-specific logic with 3–6 benign exceptions. The framework is intentionally binary at the decision level: $n_{\text{closed}} \geq 1$ classifies a variant as disruptive.

S1.6 Tier S gate summary

Structured-domain Tier S gates (benign = 0):

1. X→Gly (Ch03_Core): Ramachandran conformational freedom explosion in buried sites
2. Pro→X (Ch05_Loop): Ramachandran constraint release in structured regions
3. β-branch loss in β-strand (Ch03_Core): interlock collapse in buried β-sheet
4. Surface salt bridge (Ch09_SaltBridge): electrostatic zipper disruption at surface-exposed charged pairs
5. Polar→hydrophobic buried (Ch03_Core): hydrogen-bond partner loss in deeply buried sites
6. Met sulfur network (Ch03_Core): chalcogen network disruption near Zn coordination (seismic isolation)

7. Rigid hub ≥ 2 chains (Ch08_Oligomer): tetramer-interface rigidity at multi-chain contact positions

IDR hard constraints (1D physics, no coordinates):

8. Coupled folding (Ch10_SLiM Gate C, 6-partner union): non-conservative substitution at any union-face residue = CLOSED

S/T -P kinase motif (Ch07_PTM): Pro ring loss abolishes kinase substrate recognition

9. PPII spacer β-branch (Ch10_SLiM): $\{A,G\} \rightarrow \{V,I,T\}$ restricts φ-angle in PPII helix

10. IDR Gly→X (Ch12_IDR_Gly): side-chain introduction constrains functionally required backbone freedom

11. NLS charge loss (Ch10_SLiM): basic→neutral at importin-α recognition positions

Geta layer (post-closure exceptions, both transferable across proteins):

13. Geta_VI: V↔I in buried β-branched positions (acts on Ch03_Core); rescues 0 ClinVar Pathogenic variants.

14. Geta_IDR_PTM: charge-preserving substitutions K↔R, R↔H, S↔T within ± 1 of a PTM site in IDR (acts on Ch07_PTM IDR sub-gates); rescues 0 ClinVar Pathogenic variants.

The "rescues 0 ClinVar Pathogenic variants" condition was used as a post hoc safeguard against reversing known disruptive variants, not as a fitting objective or threshold-optimization step. Geta admission requires (i) protein-independent physicochemical expression, (ii) zero ClinVar Pathogenic rescued, and (iii) generalization to any protein where the primary gate applies.

S1.7 Molecular mechanism versus clinical penetrance

ClinVar labels were used only for post hoc evaluation. The framework itself is built to detect molecular mechanism disruption, not to regress directly onto clinical penetrance. This distinction is especially important in intrinsically disordered regions, where some ClinVar-benign variants can still have explicit molecular rationales for functional disruption, such as motif destruction, PTM chemistry loss, or nuclear localization signal impairment. Accordingly, discordant calls were interpreted in two categories: likely genuine gate errors and likely true molecular positives whose molecular disruption may not be fully reflected by penetrance-based clinical labeling.
