# Supplementary material for: A zero-parameter first-principles gate framework for full-length TP53 missense variant interpretation
Source: PLoS Comput Biol. 2026 Jun 11;22(6):e1014168. doi: 10.1371/journal.pcbi.1014168 (PMC13278589; doi:10.1371/journal.pcbi.1014168)
Supplement: S2 Text — Repository structure, CLI usage (pathogenicity-gates predict, predict-batch, explain, list-proteins), input file provenance, YAML annotation schema for adding new target proteins, evaluation logic, and reproducibility notes. (PDF) [file pcbi.1014168.s008.pdf]

S2 Text

Computational implementation and reproducibility details

For: A zero-parameter first-principles gate framework for full-length TP53 missense variant interpretation

Author: Masamichi Iizumi

S2.1 Open-source package and command-line interface

The framework is distributed as the open-source Python package `pathogenicity-gates` (PyPI; v0.5.1; MIT license), installable via `pip install pathogenicity-gates`. The package provides a command-line interface with the following subcommands:

- `pathogenicity-gates predict -protein <name> -mutation <variant>` — single-variant prediction.
- `pathogenicity-gates predict-batch -protein <name> -input <CSV/JSON/TSV>` — batch prediction.
- `pathogenicity-gates explain -protein <name> -mutation <variant>` — per-channel firing diagnostics with plain-English physical mechanism descriptions.
- `pathogenicity-gates list-proteins` — list bundled protein annotations.

The package ships with bundled YAML annotations and reference structures for four proteins: p53 (this paper), KRAS, TDP-43, and BRCA1. Adding a new target protein requires only a single YAML annotation file (specifying domain ranges, IDR regions, PTM sites, partner faces if applicable, and any protein-specific motif annotations) plus a PDB or AlphaFold structure; no modification of the channel logic itself is required.

S2.2 Code and repository structure

All public code, manuscript logic files, and downloadable datasets are hosted in the GitHub repository: `https://github.com/miosync-masa/pathogenicity-gates`. The repository is archived on Zenodo with DOI: `https://doi.org/10.5281/zenodo.20492176`. Top-level directories include the installable Python package (`pathogenicity_gates/`), test suite (`tests/`), documentation (`docs/`), per-protein evaluation results (`Result/`), supplementary CSV/JSON tables (`supplementary/`), and the manuscript-ready paper material (`paper_markdown/`). The repository is public and distributed under an MIT license.

S2.3 Input files and provenance

The TP53 evaluation requires the bundled ClinVar missense classification file, PDB coordinate files (1TSR, 1YCR, 2J0Z, 5HPD, 5HOU, 2L14, 2K8F, 2MZD; available from the Protein Data Bank), and the SSOC v3.32 implementation used to derive local structural features. The 6-partner Gate C union face is precomputed and stored as `partner_face.json` in the bundled p53 data. Cross-protein evaluations (KRAS, TDP-43, BRCA1) use protein-specific bundled structural and annotation data shipped with the package; the corresponding YAML annotation files document the per-protein domain organization, motif annotations, and partner faces (where applicable).

S2.4 Evaluation logic

Variants are first filtered to the in-scope full-length regions covered by the channel set. Each variant is then evaluated against all channels relevant to its domain context. For structured regions, this includes coordinate-derived channels (Ch01–Ch09); for disordered regions, this includes IDR-specific channels (Ch10_SLiM, Ch11_IDR_Pro, Ch12_IDR_Gly) and the IDR sub-gates of Ch07_PTM. Both Getas (Geta_VI on Ch03_Core; Geta_IDR_PTM on Ch07_PTM IDR sub-gates) are applied as post-closure exceptions. A variant is predicted disruptive if at least one channel closes after the Geta layer ($n_{\text{closed}} \geq 1$). Primary evaluation metrics are computed against ClinVar Pathogenic and Benign labels, while VUS calls are reported separately as candidate disruptive variants.

S2.5 Reproducibility notes

The framework is deterministic and rule-based. Reproducibility therefore depends on version-locking the channel definitions, the amino-acid property tables, the structural input files, the in-scope variant list, and the partner-face JSON. Because the decision rule is binary and non-fitted, any change in outputs should be traceable to a changed input file, an altered gate definition, or a scope correction rather than to stochastic optimization. The archived Zenodo release (DOI: `https://doi.org/10.5281/zenodo.20492176`) and the PyPI release of `pathogenicity-gates v0.5.1` provide the version-locked snapshot used for the results reported in the main text. The CLI run log is reproduced byte-for-byte by:

```
pip install pathogenicity-gates==0.5.1
pathogenicity-gates predict-batch --protein p53 \
    --input <bundled tp53_clinvar_missense.csv> \
    --output v18_final_results.json
```
