# Supplementary material for: A zero-parameter first-principles gate framework for full-length TP53 missense variant interpretation
Source: PLoS Comput Biol. 2026 Jun 11;22(6):e1014168. doi: 10.1371/journal.pcbi.1014168 (PMC13278589; doi:10.1371/journal.pcbi.1014168)
Supplement: S1 — Repository manifest. Complete manifest of the GitHub repository, including bundled YAML annotations and reference structures for p53, KRAS, TDP-43, and BRCA1. (PDF) [file pcbi.1014168.s009.pdf]

S1 Dataset

Repository manifest and public data/code availability

For: A zero-parameter first-principles gate framework for full-length TP53 missense variant interpretation

Author: Masamichi Iizumi

Repository: `https://github.com/miosync-masa/pathogenicity-gates`

PyPI package: `pathogenicity-gates` v0.5.1 (`pip install pathogenicity-gates`)

Archived version: Zenodo DOI: `https://doi.org/10.5281/zenodo.20492176`

License: MIT

Repository components:

- `pathogenicity_gates/` — installable Python package (channel and Geta logic, CLI, structure parsers, bundled data for four proteins).
- `pathogenicity_gates/data/{p53,kras,tdp43,brca1}/` — per-protein bundled YAML annotations, reference structures, PTM annotations, and partner-face JSON files.
- `tests/` — pytest suite covering channel logic, CLI commands, and per-protein evaluation.
- `Result/` — per-variant prediction outputs, gate-firing logs, and run logs (including `v18_final_results_dump.json`).
- `supplementary/` — machine-readable CSV/JSON tables corresponding to S1–S6 Tables of this manuscript.
- `paper_markdown/` — design rationale, transferability matrix, and per-channel physical justification.
- `README.md` — repository overview and usage instructions.
- `LICENSE` — MIT license.
