# Supplementary material for: A zero-parameter first-principles gate framework for full-length TP53 missense variant interpretation
Source: PLoS Comput Biol. 2026 Jun 11;22(6):e1014168. doi: 10.1371/journal.pcbi.1014168 (PMC13278589; doi:10.1371/journal.pcbi.1014168)
Supplement: Attachment — Submitted filename: Response to Reviewers.pdf [file pcbi.1014168.s010.pdf]

Response to Reviewers

Manuscript ID: PCOMPBIOL-D-26-00680 **Title:** A zero-parameter first-principles gate framework for full-length TP53 missense variant interpretation **Authors:** Masamichi Iizumi

---

Letter to the Editor

Dear Dr. Najmanovich and Dr. Ben-Tal,

We thank the Academic Editor and Reviewer #1 for their thoughtful and constructive evaluation of our manuscript. We were genuinely encouraged by the recognition that the framework "does not suffer of any methodological pitfalls, nor does it reach beyond what it claims to achieve," and by Reviewer #1's remark that the contribution "is of very high quality and absolutely [sound] in all aspects of the fundamentals [of how] missense variants can affect the structure and the function of proto-oncogenes and tumor suppressors."

We are particularly grateful for the central charge raised by both reviewers: to make the framework not merely a TP53 case study but a foundation that other investigators can build upon for other proteins. We took this as an invitation to demonstrate, rather than merely claim, that the architecture is transferable. We have responded with three substantive changes, supported by implementation infrastructure that we had not adequately emphasized in the original submission.

Summary of revisions

1. **Framework upgrade: v17 → v18 FINAL.** Reviewer #1 explicitly asked whether the p53 TAD × CBP TAZ2 complex is incorporated in the coupled-folding gate. *In v17, it was not.* Recognizing this as exactly the kind of "missing gate" our framework is designed to surface, we expanded Gate C from a 1-partner face (MDM2 only, 1YCR, 11 residues) to a 6-partner union (MDM2 + CBP TAZ2/TAZ1/NCBD + p300 TAZ2 × 2; PDBs 1YCR, 5HPD, 5HOU, 2L14, 2K8F, 2MZD; 59 union residues). This single architectural change yielded:

- **TAD1 sensitivity: 57.9% → 92.1% (+34.2 pp)**
- **TAD2 sensitivity: 23.8% → 76.2% (+52.4 pp)**
- **Overall sensitivity: 80.8% → 84.5% (+3.7 pp)**
- **Core sensitivity preserved at 90.9% (no regression)**
- **PPV preserved at 89.1%**

We respectfully wish to acknowledge Reviewer #1 in this regard: the question itself diagnosed the missing physics that drove the largest single improvement in this revision.

2. **New architecture: Hierarchical Gate-Geta layer.** The 6-partner Gate C expansion was introduced together with two "Geta" rescue rules — physically grounded post-closure exceptions that prevent legitimate-tolerance variants from being miscalled when a primary gate closes. The framework now consists of **12 channels and 2 Getas** at the top level (Ch01–Ch12 in the CLI implementation; Geta_VI for V↔I isomeric swaps in buried β-branched positions; Geta_IDR_PTM for charge- preserving substitutions adjacent to PTM sites in IDR regions). Both Getas are validated against the entire ClinVar Pathogenic set: zero Pathogenic variants are rescued by either Geta. This formalizes a key methodological commitment: *do not lower thresholds; add structure*. A new Methods subsection ("Hierarchical Gate-Geta Architecture") describes the layer.
3. **Generalization beyond TP53.** We have added a new Discussion subsection ("Generalization to other proteins") together with a new Table 12 (per-variant cross-protein outcome on KRAS, TDP-43, and BRCA1) and a new Figure 8 (cross-protein validation visual). The transferability classification (UNIVERSAL / ADAPTABLE / SPECIFIC) of all 12 channels and 2 Getas is given in the Discussion text and as a machine-readable S5 Table. The framework is now distributed as the open-source Python package `pathogenicity-gates` (v0.5.1, MIT-licensed, `pip install pathogenicity-gates`), with a command-line interface and bundled annotations for four proteins (p53, KRAS, TDP-43, BRCA1). Adding a new target protein requires only a YAML annotation file and a PDB or AlphaFold structure.

We hope these changes directly address the central concern shared by the Editor and Reviewer #1, while preserving the core philosophical commitment of the framework — namely, that thresholds are derived from physics rather than fitted to

data, and that missed variants are evidence of missing physical mechanisms rather than evidence that thresholds are too strict.

We thank the editorial office for the opportunity to revise.

Sincerely, Masamichi Iizumi Miosync, Inc., Tokyo, Japan

Detailed Response to Academic Editor

Editor (Najmanovich): *"It will be important to expand the work to include a thorough discussion of what channels/rules are applicable to other proteins. Of course it would be a tremendous task to create a framework applicable to all proteins and this is not what is being asked of the author — but a discussion of what subset of channels, and perhaps even the creation of code applicable to other soluble proteins at least, will create the foundation for the future expansion of this framework."*

We have responded along three axes: a transferability classification, a cross-protein validation, and a publicly distributed implementation.

(E1) Transferability classification

In the revised Discussion section "Generalization to other proteins," we classify each of the 12 channels and 2 Getas in v18 FINAL into three transferability tiers:

- **UNIVERSAL (5 channels + 2 Getas):** applicable to any protein from amino-acid sequence and standard functional annotations (IDR / PTM / SLiM) alone. These include Ch07_PTM (PTM site and proximity logic; including the proline-directed [S/T]-P kinase sub-gate), Ch10_SLiM (motif disruption and the architectural pattern of Gate C), Ch11_IDR_Pro (context-dependent proline logic in disordered regions), Ch12_IDR_Gly (the IDR glycine constraint, which formalizes that the absence of a side chain can itself be functional), and the SLiM identity sub-rules within Ch10. Both Getas (Geta_VI for V↔I exchange in any buried β-branched position; Geta_IDR_PTM for charge-preserving substitutions K↔R, R↔H, S↔T adjacent to PTM sites in IDR regions) are protein- independent by construction.

- **ADAPTABLE (5 channels):** require structural context that can be satisfied either by an experimental PDB *or* by an AlphaFold prediction with pLDDT > 70 (a regime in which predicted and experimental backbones are effectively interchangeable for burial, secondary structure, and contact geometry). These include Ch03_Core (cavity, steric, polarity, hydrogen-bond, and charge integrity in buried sites), Ch04_SS (secondary structure propensity and backbone strain), Ch05_Loop (Ramachandran endpoint logic for Gly and Pro), Ch06_PPI (structured-domain protein- protein interface contacts), and Ch09_SaltBridge (surface electrostatic zipper networks).
- **SPECIFIC (2 channels):** require a target-specific structural input that AlphaFold alone cannot supply. Ch01_DNA requires a DNA-bound complex (1TSR for p53); Ch02_Zn requires a known metal-binding site (the HCCC tetrahedral coordination shell in p53 core).

A critical distinction: **Gate C** (the coupled-folding sub-gate within Ch10_SLiM) is SPECIFIC in *data requirements* (it needs partner-complex structures) but UNIVERSAL in *architecture*. The "union of partner faces + non-conservative filter" pattern transfers 1:1 to any coupled-folding interaction. We demonstrate this explicitly on BRCA1 BRCT × {ABRAXAS1, BACH1, CtIP} (PDBs 1T29, 1T15, 1Y98); the same extraction pipeline applies without modification.

The transferability classification is summarized in the revised Discussion (subsection "Generalization to other proteins") and is provided as a machine-readable S5 Table in the Supplementary Information. The per-variant cross-protein outcome is given as Table 12 in the revised manuscript and as S6 Table (CSV).

(E2) Cross-protein validation

The central question — "do the p53 gates carry meaning for other proteins?" — is most directly answered by applying the same framework to additional proteins and listing, for each variant, which p53-derived gate captures it and which gates would need to be added.

We tested three additional cancer-relevant proteins. The results are summarized in new Figure 8 and new S6 Table; the full per-variant table appears below.

Protein	Variants captured by p53-derived gates	What additional gate is needed
**KRAS**	G12V/D/C, G13D, K117N, A146T, K184Q (Ch03_Core, Ch04_SS, IDR charge)	GTP-cofactor channel for Q61H/L
**TDP-43**	G294A, G295V, G348C (Ch12_IDR_Gly); A315T, A382T (Ch10 β-branch); N382D, N390D (Ch10 IDR charge)	Aggregation / LLPS channel for M337V
**BRCA1**	C61G, C64R, T37R (Ch02 Zn-analog, Ch03 charge); V1696L, K1702N, A1708E, M1775R, C1697R (Ch03 buried); S1655F, R1699Q/W, S1715R (Gate C with partners ABRAXAS1, BACH1, CtIP — PDBs 1T29, 1T15, 1Y98)	None within tested set

Three observations follow directly from this table:

1. **The p53 gates do carry meaning for other proteins.** The same physical channels — buried-site integrity (Ch03_Core), secondary-structure strain (Ch04_SS), Zn-coordination (Ch02_Zn), surface-charge logic, IDR glycine constraint (Ch12_IDR_Gly), IDR β-branch and charge logic (Ch10_SLiM), and Gate C (coupled-folding partner face) — all activate on the corresponding mechanism in KRAS, TDP-43, and BRCA1.
2. **Gate C is architecturally portable.** For BRCA1 BRCT, replacing {MDM2, CBP TAZ2/TAZ1/NCBD, p300 TAZ2} with {ABRAXAS1, BACH1, CtIP} in the partner set produces a working Gate C for the BRCT phospho- pocket variants without modifying the channel logic itself. The "union of partner faces + non-conservative filter" pattern is protein-independent.
3. **Where a gate is missing, the framework names what to add.** Q61H/L in KRAS are not captured because no gate currently encodes GTP-cofactor chemistry; M337V in TDP-43 is not captured because no gate currently encodes aggregation/LLPS physics. These are not performance failures; they are the framework's own diagnostic output, identifying the specific physical mechanism whose IF-THEN translation has not yet been written. In both cases the missing physics is well-defined and independently studied (GTPase transition-state stabilization; condensate-forming sequence grammar), so the construction of the corresponding channel is a finite translation task

scheduled for subsequent package releases, not an open conceptual question.

In short: *the p53 gates work on other proteins, and where they don't, the reason is physically explicit and the corresponding gate is a finite translation away from being written.*

(E3) Open-source implementation

The framework is now distributed as the Python package `pathogenicity-gates` (PyPI; v0.5.1; MIT license):

```
pip install pathogenicity-gates
pathogenicity-gates list-proteins
pathogenicity-gates predict --protein p53 --mutation R175H
pathogenicity-gates explain --protein brca1 --mutation C61G
pathogenicity-gates predict-batch --protein p53 ¥
    --input variants.csv --output results.json
```

The repository (https://github.com/miosync-masa/pathogenicity-gates) ships with bundled YAML annotations and reference structures for p53, KRAS, TDP-43, and BRCA1. **Adding a new target protein requires only a single YAML annotation file and a PDB (or AlphaFold) structure** — no code modification. The data schema is documented in the repository README, and a new "Code availability" section in the revised manuscript describes the package, its CLI, and the YAML annotation format.

This directly addresses the Editor's recommendation to provide "code applicable to other soluble proteins at least, [to] create the foundation for the future expansion of this framework."

Detailed Response to Reviewer #1

Reviewer #1: *"I have read the manuscript with a lot of interest and captivation. ... I find this contribution is of very high quality and absolutely [sound] in all aspects of*

the fundamentals [of how] missense variants can affect the structure and the function of proto-oncogenes and tumor suppressors."

We thank Reviewer #1 for the warm and substantive review. The three specific issues raised (missing physics including TAZ2, AlphaFold compatibility, accessibility to non-expert users) have shaped the revisions described below.

(R1.1) Missing physics — phase separation, exclusion, completeness of interactors, and TAZ2

"The list of rules in the number of channels in the V17 framework is quite exhaustive. Do the authors have possibily in mind? Failure to phase separate in proper condensates? Or to be excluded? Incomplete list of interactors? In this regard, do the structure of the complex of the p53 TAD with the TAZ2 domain of CBP (a coactivator) included in a channel like the interaction with MDM2?"

This question struck at the heart of the framework's design philosophy, and we are grateful that Reviewer #1 articulated it so precisely.

On the TAZ2 question (the immediate one): In v17, the answer was *no*. Gate C used MDM2 (1YCR) as a single-partner face. The CBP TAZ2 / TAZ1 / NCBD and p300 TAZ2 interactions were *not* incorporated. Recognizing that Reviewer #1 had identified the precise missing-physics that we ourselves had failed to include, we expanded Gate C to a 6-partner union:

PDB	Partner	TAD region
1YCR	MDM2	TAD1 (residues 17-29)
5HPD	CBP TAZ2	TAD1
5HOU	CBP TAZ1	TAD1
**2K8F**	**CBP TAZ2**	**TAD1** ← Reviewer #1's question
**2L14**	**p300 TAZ2**	**TAD2** ← Reviewer #1's question
2MZD	NCBD	TAD2

The resulting union covers 59 residues across TAD1 and TAD2. The sensitivity gain was concentrated exactly where the question predicted — at the TADs:

- **TAD1 sensitivity: 57.9% → 92.1% (+34.2 pp)**

- **TAD2 sensitivity: 23.8% → 76.2% (+52.4 pp)**

These improvements are documented in the revised Table 2. The trajectory — ″missed variant → missing gate → new PDB structure → expanded channel″ — is, within our framework, the operational expression of the design philosophy that thresholds should not be lowered to capture missed variants. Reviewer #1 walked us through one full iteration of that philosophy within the review process itself, and we make this point explicit in the revised Discussion.

On the broader question (LLPS, exclusion, completeness): Several physical regimes remain to be translated into IF-THEN gates within v18 FINAL. The cross-protein validation in (E2) makes these visible: TDP-43 M337V is not captured because no gate yet encodes aggregation/LLPS physics; KRAS Q61H/L is not captured because no gate yet encodes GTPase catalytic / cofactor chemistry; *exclusion* (the absence of an interaction whose presence would have been disruptive) is not captured because no gate yet encodes the inverse of Gate C. Within p53 itself, the same logic applies at TAD1 position 4, where P4R (Pathogenic, charge introduction) is correctly captured by Gate C while P4L and P4Q (both Benign) are also closed; together, these three variants at the same position specify a direction-aware filter (charge introduction CLOSED; hydrophobic / polar introduction OPEN at TAD boundary positions). For each of these cases, the required physical input is well-defined: aromatic spacing and charge patterning for the LLPS channel; transition-state geometry around catalytic Mg^{2+}/GTP for the catalytic channel; partner-pocket geometry for the exclusion channel; established side-chain chemistry of charge versus hydrophobic introduction for the TAD-boundary direction-aware Geta. In every case, what stands between the current framework and the captured variant is a finite IF-THEN translation of established physics, not a search for new principles. The corresponding gates are scheduled for subsequent releases of the package; the manuscript benchmark is held fixed at v0.5.1 (v18 FINAL) for reproducibility, not because their construction is an open question. We list these in a new Discussion subsection (″Next gates to be written″).

(R1.2) AlphaFold and AlphaFold-Multimer for proteins lacking experimental coverage

"Can some of the channels be based on predictions such as structural models from AlphaFold and AlphaFold Multimer when experimental structural knowledge is lacking?"

Yes, and this is now formalized in the transferability classification (see E1 above and a new Methods subsection "AlphaFold compatibility"). Briefly:

- **Structured-domain channels (Ch03_Core, Ch04_SS, Ch05_Loop, Ch06_PPI, Ch09_SaltBridge):** These compute burial, secondary structure, contact density, and charge-network proximity from coordinates. For protein regions with AlphaFold pLDDT > 70, predicted backbones are effectively interchangeable with experimental ones for these geometric quantities. We document this threshold and the underlying reasoning in the revised Methods.
- **IDR channels (Ch10_SLiM, Ch11_IDR_Pro, Ch12_IDR_Gly, Ch07_PTM IDR sub-gates):** These are one-dimensional and operate on sequence and annotation alone — no coordinates required. AlphaFold's well-known weakness in IDRs (pLDDT < 50) is therefore *not* a limitation of our framework in IDR regions. The 1D design of these channels was chosen precisely to bypass this limitation.
- **Coupled-folding interfaces (Gate C within Ch10_SLiM):** AlphaFold- Multimer is the natural way to extend Gate C to partners without experimental complex structures. The interface-residue-extraction pipeline that produces the union face accepts either experimental or AlphaFold-Multimer predictions, with appropriate confidence filters. We note that experimental complexes remain preferred where available because the partner identity itself (rather than the geometry alone) is biologically meaningful.

A concise summary of which channels are AlphaFold-compatible appears in the revised Methods (subsection "AlphaFold compatibility for proteins lacking experimental coverage") and in the transferability matrix (S5 Table).

(R1.3) Accessibility to investigators not expert in physicochemistry, and integration with ML approaches

"Users may not be as expertly versed and aware of all the physicochemical and structural determinants of protein stability, molecular recognition, PTM and polymer physics as the authors. It is not clear to me how both approaches [physics-based

and ML-based] can or could be used in an integrated fashion. Maybe the authors can expand in this perspective."

We have addressed accessibility through the open-source CLI implementation described in (E3) above. The end-user does not need to know the internal physics of any channel: a single-line invocation returns both the prediction and a per-channel firing breakdown that names the physical mechanism in plain English. For example,

```
$ pathogenicity-gates explain --protein p53 --mutation R175H
  Prediction: Pathogenic (n_closed = 3)
  Ch02_Zn:        CLOSED  - direct coordination ligand disrupted
  Ch03_Core:      CLOSED  - buried steric/charge perturbation
  Ch05_Loop:      CLOSED  - Ramachandran constraint violated
  (other channels: OPEN)
```

This makes the framework usable by structural biologists, clinicians, and computational biologists who are not specialists in any one of the underlying physical regimes.

On the integration with machine-learning approaches, we have added a paragraph to the Discussion ("Complementarity with regression-based predictors"). We view Gate & Channel as a *complement to*, not a replacement for, fitted predictors:

- ML predictors excel at integrating weak, high-dimensional statistical signal across very large datasets and produce smooth, well-calibrated rankings.
- Gate & Channel produces sparse, auditable, mechanistic calls and names the physical violation behind each call.
- A natural integration pattern is *physics-first triage*: variants that fire any Tier-S gate (Ch02_Zn and Ch09_SaltBridge in p53 each have PPV = 100%) carry high mechanistic priority by themselves; variants that fire no gate can be passed to a regression-based predictor for ranking; mid-confidence variants benefit from both views being available simultaneously.

We do not present a specific integrated tool in this paper, but the CLI output format is explicitly designed to be machine-readable so that downstream integration is straightforward.

(R1.4) Closing acknowledgment

We are particularly grateful for Reviewer #1's TAZ2 question. Within our framework, that question *was* the missing gate; the v17 → v18 expansion is, in a precise sense, the framework operating as designed under external diagnosis. This is exactly the kind of dialogue we hoped the manuscript would invite, and we thank Reviewer #1 for engaging at precisely that level.

Response to Journal Requirements

1. **LaTeX source file:** Provided as `main_v18.tex` (uploaded as item type "LaTeX Source File"); the compiled PDF is uploaded as "Manuscript."
2. **Figure formats:** All figures are provided as separate `.eps` files (Fig1.eps – Fig8.eps), processed via PLOS's NAAS tool prior to upload.
3. **Data availability:** The full ClinVar-derived dataset, gate-level prediction outputs for all 1,369 in-scope variants, per-region breakdowns, and per-protein transferability data are available without restriction at the GitHub repository (https://github.com/miosync-masa/pathogenicity-gates) and the Zenodo archive (DOI: 10.5281/zenodo.19214741). The minimal dataset underlying every figure and table is included in the repository's `Result/` and `supplementary/` directories. The pip-installable Python package `pathogenicity-gates` (v0.5.1) reproduces all main-text numerical results from the bundled inputs.

Confusion-matrix summary (v17 → v18 FINAL)

For convenience, the headline numerical changes (all reproducible via the CLI, `pathogenicity-gates predict-batch --protein p53`):

Metric	v17 (original submission)	v18 FINAL (this revision)	Δ
TP	523	**547**	+24
FP	64	67	+3
FN	124	**100**	−24

Metric	v17 (original submission)	v18 FINAL (this revision)	Δ
TN	70	67	−3
Sensitivity	80.8%	**84.5%**	+3.7 pp
Specificity	52.2%	50.0%	−2.2 pp
PPV	89.1%	**89.1%**	±0
NPV	36.1%	40.1%	+4.1 pp
Hotspots captured	9 / 9	9 / 9	—
Fitted parameters	0	0	—

Per-region ΔSensitivity (v17 → v18 FINAL):

Region	v17	v18 FINAL	Δ
TAD1 (1–40)	57.9%	**92.1%**	**+34.2 pp**
TAD2 (41–61)	23.8%	**76.2%**	**+52.4 pp**
PRD (62–93)	66.7%	66.7%	±0
Core (94–289)	90.9%	90.9%	±0
Linker (290–324)	41.9%	41.9%	±0
Tet (325–356)	67.5%	67.5%	±0
CTD (357–393)	70.6%	70.6%	±0

The improvements are concentrated entirely in TAD1 and TAD2 — the regions that contain the coupled-folding interfaces with MDM2, CBP, p300, and NCBD. This is the precise localization that Reviewer #1's TAZ2 question predicted.

List of changes (for marked-up version)

The marked-up version highlights the following additions and modifications:

- **Abstract:** updated headline numbers (v17 → v18 FINAL); one new sentence on the Gate-Geta architecture and CLI distribution.
- **Introduction:** unchanged in structure; minor updates in last paragraph.
- **Methods, new subsection:** "Hierarchical Gate-Geta Architecture."

- **Methods, new subsection:** ″AlphaFold compatibility.″
- **Methods, channel naming:** unified to CLI naming (Ch01–Ch12).
- **Results, Section 3.1:** all numerical values updated to v18 FINAL.
- **Results, Section 3.2:** Gate C 6-partner expansion described.
- **Results, new Section 3.4:** ″Cross-protein validation: KRAS, TDP-43, BRCA1.″
- **Discussion, new subsection:** ″Generalization to other proteins.″
- **Discussion, new subsection:** ″Missing-physics frontiers.″
- **Discussion, new subsection:** ″Complementarity with regression-based predictors.″
- **New Table 12:** Cross-protein per-variant outcome (KRAS, TDP-43, BRCA1).
- **New S5 Table:** Transferability matrix (12 channels + 2 Getas × 3 tiers).
- **New S6 Table:** Cross-protein gate firing (per-variant, machine-readable).
- **New Figure 7:** Hierarchical Gate-Geta architecture (concept diagram).
- **New Figure 8:** Cross-protein validation (KRAS, TDP-43, BRCA1).
- **New SI section S7 Text:** Generalization to other proteins (extended).
- **New S5 Table:** Transferability matrix (machine-readable CSV).
- **New S6 Table:** Cross-protein gate firing (machine-readable CSV).
- **New ″Code availability″ section:** describes pathogenicity-gates v0.5.1 package, CLI, and YAML schema.

— END OF RESPONSE TO REVIEWERS —
