# Supplementary material for: A zero-parameter first-principles gate framework for full-length TP53 missense variant interpretation
Source: PLoS Comput Biol. 2026 Jun 11;22(6):e1014168. doi: 10.1371/journal.pcbi.1014168 (PMC13278589; doi:10.1371/journal.pcbi.1014168)
Supplement: Peer review history

# Peer review: Decision Letter 0

**DOI:** 10.1371/journal.pcbi.1014168.r001

**Authors:** Nir Ben-Tal, Rafael Najmanovich

21 Apr 2026

PCOMPBIOL-D-26-00680

A zero-parameter first-principles gate framework for full-length TP53 missense variant interpretation

PLOS Computational Biology

Dear Dr. iizumi,

Thank you for submitting your manuscript to PLOS Computational Biology. After careful consideration, we feel that it has merit but does not fully meet PLOS Computational Biology's publication criteria as it currently stands. Therefore, we invite you to submit a revised version of the manuscript that addresses the points raised during the review process.

Please submit your revised manuscript by Jun 21 2026 11:59PM. If you will need more time than this to complete your revisions, please reply to this message or contact the journal office at ploscompbiol@plos.org. When you're ready to submit your revision, log on to https://www.editorialmanager.com/pcompbiol/ and select the 'Submissions Needing Revision' folder to locate your manuscript file.

Please include the following items when submitting your revised manuscript:

* A letter that responds to each point raised by the editor and reviewer(s). You should upload this letter as a separate file labeled 'Response to Reviewers'. This file does not need to include responses to formatting updates and technical items listed in the 'Journal Requirements' section below.

* A marked-up copy of your manuscript that highlights changes made to the original version. You should upload this as a separate file labeled 'Revised Manuscript with Track Changes'.

* An unmarked version of your revised paper without tracked changes. You should upload this as a separate file labeled 'Manuscript'.

If you would like to make changes to your financial disclosure, competing interests statement, or data availability statement, please make these updates within the submission form at the time of resubmission. Guidelines for resubmitting your figure files are available below the reviewer comments at the end of this letter.

We look forward to receiving your revised manuscript.

Kind regards,

Rafael J. Najmanovich

Academic Editor

PLOS Computational Biology

Nir Ben-Tal

Section Editor

PLOS Computational Biology

**Additional Editor Comments:**

This article was carefully reviewed by one invited reviewer as well as myself, the editor. Both myself and the reviewer are of the opinion that this article do not suffer of any methodological pitfalls, nor does it reach beyond what it claims to achieve. Namely, to create a framework with which to understand based on physical factors, as opposed to a machine learning framework, the effect of mutations on P53. The reviewers concerns as well as my own are the same: Whereas the proposed framework is intuitive and exhaustive, in the manner in which it is presented, it is uniquely applicable to this one specific protein. Considering the extensive existing data on the effect of mutations on P53, it is unclear how useful is this methodology, even to understand P53 alone, given the amount of existing data on it. Yet, as I believe it to be important to foster diversity of approaches in an AI dominated scientific landscape, particularly approaches that strive to provide clear physics/chemistry based explanations, I believe that this is a contribution worth of publication at Plos Computational Biology. However, it will be important to expand the work to include a thorough discussion of what channels/rules are applicable to other proteins. Of course it would a tremendous task to create a framework applicable to all proteins and this is not what is being asked of the author - but a discussion of what subset of channels, and perhaps even the creation of code applicable to other soluble proteins at least, will create the foundation for the future expansion of this framework. Essentially, what I ask is the author to add a section and ideally but not necessarily code, that goes in great detail about what channels can be used for other proteins and in what manner, to make this article relevant for those studying other proteins or wishing to work on the this framework as a foundation.

**Journal Requirements:**

If the reviewer comments include a recommendation to cite specific previously published works, please review and evaluate these publications to determine whether they are relevant and should be cited. There is no requirement to cite these works unless the editor has indicated otherwise.

1) We ask that a manuscript source file is provided at Revision. Please upload your manuscript file as a .doc, .docx, .rtf or .tex. If you are providing a .tex file, please upload it under the item type u2018LaTeX Source Fileu2019 and leave your .pdf version as the item type u2018Manuscriptu2019.

2) Please upload all main figures as separate Figure files in .tif or .eps format. For more information about how to convert and format your figure files please see our guidelines:

https://journals.plos.org/ploscompbiol/s/figures

3) Thank you for stating "ClinVar variant classifications used for evaluation are available from the NCBI ClinVar database (https://www.ncbi.nlm.nih.gov/clinvar/)." Please note that, though access restrictions are acceptable now, your entire minimal dataset will need to be made freely accessible if your manuscript is accepted for publication. This policy applies to all data except where public deposition would breach compliance with the protocol approved by your research ethics board. If you are unable to adhere to our open data policy, please kindly revise your statement to explain your reasoning and we will seek the editor's input on an exemption.

**Reviewers' comments:**

Reviewer's Responses to Questions

**Comments to the Authors:**

**Please note here if the review is uploaded as an attachment.**

Reviewer #1: I have read the manuscript with a lot of interest and captivation. My review focuses on the structural biology aspect of the work.

Generally, this work is timely and invites the structural and cancer biology fields as well as clinicians to reflect on the scientific value and accuracy of the prediction of oncogenic (pathogenic) variants of proto-oncogenes and tumor suppressors (alike TP53) existing deep leaning approaches prediction of the pathogenicity of missense variants. These are based on large databases and optimization of weight that factor recurrent patterns to achieve prediction. Whereas these are performing well, the optimized weight do not inform us on the exact molecular etiology or physicochemical origin of the disruption of the normal or WT molecular function. In this context and to complement (or rationalize) machine leaning approaches XX et al. propose a simple computational framework that is called Gate and Channel. Each channel represent a molecular or physical mechanism of disruption of function caused by the variant residues: cavity formation or clash in folded domains, mutation at the interface of the tetramerization domain and MDM2

The failure to close a gate or to identify pathogenic variant is interpreted as a lack of “physics” or a rule in a channel. The list of rules in the number of channels in the V17 framework is quite exhaustive. Do the authors have possibily in mind? Failure to phase separate in proper condensates? Or to be excluded? Incomplete list of interactors? In this regard, do the structure of the complex of the p53 TAD with the TAZ2 domain of CBP (a coactivator) included in a channel like the interaction with MDM2?

Although I completely concur with the necessity of understanding the etiology of pathogenic mutations as well as complementing the existing machine learning approaches, I am not sure how this framework can be deployed at large. P53 is one of the most structurally and mechanistically studied tumor suppressor. This is certainly contributing to the actual success of the approach. However, not all proto-oncogenes and tumor suppressors have attracted the same scrutiny and annotations. Can some of the channels be based on predictions such as structural models from AlphaFold and AlphaFold Multimer when experimental structural knowledge is lacking? In addition, users may not be as expertly versed and aware of all the physicochemical and structural determinants of protein stability, molecular recognition, PTM and polymer physics as the authors. It is not clear to me how both approaches can or could be used in an integrated fashion. Maybe the authors can expand in this perspective.

Notwithstanding these comments, I find this contribution is of very high quality and absolutely found in all aspects of the fundamentals missense variants can affect the structure and the function of proto-oncogenes and tumor suppressors.

**********

**Have the authors made all data and (if applicable) computational code underlying the findings in their manuscript fully available?**

The PLOS Data policy requires authors to make all data and code underlying the findings described in their manuscript fully available without restriction, with rare exception (please refer to the Data Availability Statement in the manuscript PDF file). The data and code should be provided as part of the manuscript or its supporting information, or deposited to a public repository. For example, in addition to summary statistics, the data points behind means, medians and variance measures should be available. If there are restrictions on publicly sharing data or code —e.g. participant privacy or use of data from a third party—those must be specified.

Reviewer #1: Yes

**********

PLOS authors have the option to publish the peer review history of their article (what does this mean?). If published, this will include your full peer review and any attached files.

If you choose “no”, your identity will remain anonymous but your review may still be made public.

**Do you want your identity to be public for this peer review?**  For information about this choice, including consent withdrawal, please see our Privacy Policy.

Reviewer #1: No

**Figure resubmission:**

While revising your submission, we strongly recommend that you use PLOS’s NAAS tool (https://ngplosjournals.pagemajik.ai/artanalysis) to test your figure files. NAAS can convert your figure files to the TIFF file type and meet basic requirements (such as print size, resolution), or provide you with a report on issues that do not meet our requirements and that NAAS cannot fix.

After uploading your figures to PLOS’s NAAS tool - https://ngplosjournals.pagemajik.ai/artanalysis, NAAS will process the files provided and display the results in the "Uploaded Files" section of the page as the processing is complete. If the uploaded figures meet our requirements (or NAAS is able to fix the files to meet our requirements), the figure will be marked as "fixed" above. If NAAS is unable to fix the files, a red "failed" label will appear above. When NAAS has confirmed that the figure files meet our requirements, please download the file via the download option, and include these NAAS processed figure files when submitting your revised manuscript.

**Reproducibility:**

To enhance the reproducibility of your results, we recommend that authors of applicable studies deposit laboratory protocols in protocols.io, where a protocol can be assigned its own identifier (DOI) such that it can be cited independently in the future. Additionally, PLOS ONE offers an option to publish peer-reviewed clinical study protocols. Read more information on sharing protocols at https://plos.org/protocols?utm_medium=editorial-email&utm_source=authorletters&utm_campaign=protocols

# Author comment: Author response to Decision Letter 1

**DOI:** 10.1371/journal.pcbi.1014168.r002

7 May 2026

Attachment Submitted filename: Response to Reviewers.pdf

# Editor report: Decision Letter 1

**DOI:** 10.1371/journal.pcbi.1014168.r003

**Authors:** Nir Ben-Tal, Rafael Najmanovich

27 May 2026

Dear Mr. iizumi,

We are pleased to inform you that your manuscript 'A zero-parameter first-principles gate framework for full-length TP53 missense variant interpretation' has been provisionally accepted for publication in PLOS Computational Biology.

Before your manuscript can be formally accepted you will need to complete some formatting changes, which you will receive in a follow up email. A member of our team will be in touch with a set of requests.

Please note that your manuscript will not be scheduled for publication until you have made the required changes, so a swift response is appreciated.

IMPORTANT: The editorial review process is now complete. PLOS will only permit corrections to spelling, formatting or significant scientific errors from this point onwards. Requests for major changes, or any which affect the scientific understanding of your work, will cause delays to the publication date of your manuscript.

Should you, your institution's press office or the journal office choose to press release your paper, you will automatically be opted out of early publication. We ask that you notify us now if you or your institution is planning to press release the article. All press must be co-ordinated with PLOS.

Thank you again for supporting Open Access publishing; we are looking forward to publishing your work in PLOS Computational Biology.

Best regards,

Rafael J. Najmanovich

Academic Editor

PLOS Computational Biology

Nir Ben-Tal

Section Editor

PLOS Computational Biology

***********************************************************

The authors satisfactorily addressed all concerns from the reviewer and editor. The paper is acceptable for publication.

# Editor report: Acceptance letter

**DOI:** 10.1371/journal.pcbi.1014168.r004

**Authors:** Nir Ben-Tal, Rafael Najmanovich

PCOMPBIOL-D-26-00680R1

A zero-parameter first-principles gate framework for full-length TP53 missense variant interpretation

Dear Dr iizumi,

I am pleased to inform you that your manuscript has been formally accepted for publication in PLOS Computational Biology. Your manuscript is now with our production department and you will be notified of the publication date in due course.

The corresponding author will soon be receiving a typeset proof for review, to ensure errors have not been introduced during production. Please review the PDF proof of your manuscript carefully, as this is the last chance to correct any errors. Please note that major changes, or those which affect the scientific understanding of the work, will likely cause delays to the publication date of your manuscript.

Soon after your final files are uploaded, unless you have opted out, the early version of your manuscript will be published online. The date of the early version will be your article's publication date. The final article will be published to the same URL, and all versions of the paper will be accessible to readers.

For Research, Software, and Methods articles, you will receive an invoice from PLOS for your publication fee after your manuscript has reached the completed accept phase. If you receive an email requesting payment before acceptance or for any other service, this may be a phishing scheme. Learn how to identify phishing emails and protect your accounts at https://explore.plos.org/phishing.

Thank you again for supporting PLOS Computational Biology and open-access publishing. We are looking forward to publishing your work!

With kind regards,

Judit Kozma

PLOS Computational Biology | Carlyle House, Carlyle Road, Cambridge CB4 3DN | United Kingdom ploscompbiol@plos.org | Phone +44 (0) 1223-442824 | ploscompbiol.org | @PLOSCompBiol
